# *Slc20a1/Pit1* and *Slc20a2/Pit2* are essential for normal skeletal myofiber function and survival

**DOI:** 10.1038/s41598-020-59430-4

**Published:** 2020-02-20

**Authors:** Sampada Chande, Daniel Caballero, Bryan B. Ho, Jonathan Fetene, Juan Serna, Dominik Pesta, Ali Nasiri, Michael Jurczak, Nicholas W. Chavkin, Nati Hernando, Cecilia M. Giachelli, Carsten A. Wagner, Caroline Zeiss, Gerald I. Shulman, Clemens Bergwitz

**Affiliations:** 1 Department of Internal Medicine, Section Endocrinology, Yale University School of Medicine, New Haven, CT USA; 2 Department of Cellular&Molecular Physiology, Yale University School of Medicine, New Haven, CT USA; 3 Comparative Medicine, Yale University School of Medicine, New Haven, CT USA; 4 Department of Medicine, Division of Endocrinology, University of Pittsburgh, University of Washington, Box 355061, Foege Hall Seattle, WA 98195 USA; 5 German Diabetes Center, Düsseldorf, Germany, University of Washington, Box 355061, Foege Hall Seattle, WA 98195 USA; 6 Department of Bioengineering, University of Washington, Box 355061, Foege Hall Seattle, WA 98195 USA; 7 Institute of Physiology, University of Zürich, Switzerland and National Center of Competence in Research NCCR Kidney.CH, Zürich, Switzerland

**Keywords:** Endocrine system and metabolic diseases, Phosphorus metabolism disorders

## Abstract

Low blood phosphate (Pi) reduces muscle function in hypophosphatemic disorders. Which Pi transporters are required and whether hormonal changes due to hypophosphatemia contribute to muscle function is unknown. To address these questions we generated a series of conditional knockout mice lacking one or both house-keeping Pi transporters *Pit1* and *Pit2* in skeletal muscle (sm), using the postnatally expressed *human skeletal actin*-cre. Simultaneous conditional deletion of both transporters caused skeletal muscle atrophy, resulting in death by postnatal day P13. *smPit1*^−/−^*, smPit2*^−/−^ and three allele mutants are fertile and have normal body weights, suggesting a high degree of redundance for the two transporters in skeletal muscle. However, these mice show a gene-dose dependent reduction in running activity also seen in another hypophosphatemic model (*Hyp* mice). In contrast to *Hyp* mice, grip strength is preserved. Further evaluation of the mechanism shows reduced ERK1/2 activation and stimulation of AMP kinase in skeletal muscle from *smPit1*^−/−^; *smPit2*^−/−^ mice consistent with energy-stress. Similarly, C2C12 myoblasts show a reduced oxygen consumption rate mediated by Pi transport-dependent and ERK1/2-dependent metabolic Pi sensing pathways. In conclusion, we here show that *Pit1* and *Pit2* are essential for normal myofiber function and survival, insights which may improve management of hypophosphatemic myopathy.

## Introduction

Inorganic phosphate (Pi) is involved in various cellular processes including DNA and cell membrane synthesis, signal transduction, ATP production, and bone mineralization. Serum Pi is regulated by a hormonal bone-parathyroid-kidney axis consisting of fibroblast growth factor 23 (FGF23), parathyroid hormone (PTH), and 1,25(OH)_2_-D (calcitriol)^1^. Familial disorders of Pi homeostasis are caused by mutations in components of this axis that either directly or indirectly (via homeostatic mechanisms) lower serum Pi levels. Furthermore, chronic hypophosphatemia is observed in the often vitamin D-, and therefore Pi-, deficient elderly population^2^.

How muscle weakness develops in these hypophosphatemic conditions, which Pi transporters are involved and whether the homeostatic hormonal changes, which develop as a result of hypophosphatemia, contribute to reduced muscle function is poorly understood. Previous studies suggest that decreased ATP^3,4^ and phosphodiesters^5^ may in part explain the muscle weakness seen in hypophosphatemia. We recently reported that hypophosphatemic myopathy goes along with reduced ATP flux (V_ATP_) and intracellular Pi in an individual with hereditary hypophosphatemic rickets with hypercalciuria (HHRH) and in the sodium-Pi co-transporter *Npt2a* null mouse model of this disorder^6^. Basal and insulin-stimulated muscle V_ATP_ and Pi uptake have furthermore been shown to be decreased in the offspring of patients with type 2 diabetes^7^.

While these studies support a direct role for Pi as a substrate for mitochondrial ATP production, it is not clear whether extracellular Pi needs to enter skeletal muscle to improve V_ATP_ and muscle function, and which Pi transporters and intracellular signaling pathways are involved. *Pit1 and Pit2* are type III sodium-Pi co-transporters encoded by *Slc20a1* and *Slc20a2*^8^. Initially discovered as retroviral receptors^9,10^, they since have been shown to be ubiquitously expressed Pi-transporters in mammals. Pi-uptake via *Pit1* has been shown to maintain ATP levels in pre-osteoblasts^11^ and chondrocytes^12^, and it is possible that *Pit1* has similar functions in other tissues, including skeletal muscle. In support of a role for *Pit1* in skeletal muscle ATP production, *Pit1* and Pi levels appear to correlate with fiber-type, with slow twitch having greater levels of *Pit1* and Pi than fast twitch fibers^13,14^, perhaps because slow twitch fibers require more Pi transport to maintain their high oxidative capacity. However, no skeletal muscle phenotype has been described thus far in mice with global ablation of *Slc20a1/Pit1*, which causes embryonic lethality due to liver and hematopoetic abnormalities^15^. Likewise, mice with global ablation of *Slc20a2/Pit2* are fertile, appear to thrive normally and have comparable body weights without reported skeletal muscle phenotypes^16–19^. Interestingly, recent evidence suggests that *Pit1* can stimulate cell proliferation^20^, gene expression^11^, apoptosis^21^ and activation of the mitogen-activated kinases ERK1/2 in many cells including myocytes^11,22^ independently of its transport activity. MAPK phosphatases (MKPs) can both positively and negatively regulate myogenesis through coordinate MAPK dephosphorylation^23–26^. For example, p38 MAPK is believed to be a promyogenic MAPK, while ERK1/2 has been shown to exhibit both positive and negative regulatory roles in myogenesis, and c-Jun NH2 terminus kinases (JNK) appears in some cases to be either dispensable or negative in myogenesis. Furthermore, muscle ablation of ERK1/2 isoforms using *HSA-Cre* results in severe atrophy of the soleus muscles, revealing their important role for the maintenance of myofibers and neuromuscular synapses in adult mice, particularly in type 1 slow twitch fibers^27,28^.

Also, it is not clear whether FGF23, PTH, and 1,25(OH)_2_-D can contribute to hypophosphatemic myopathy by directly affecting muscle function in a Pi transport-independent fashion. For instance, loss-of-function mutations in the sodium-Pi transport protein 2c (*SLC34A3*) gene have been associated with hypophosphatemic rickets with hypercalciuria (HHRH)^29^, a disorder that causes renal Pi wasting, rickets and kidney stones, but muscle weakness is less pronounced in these individuals when compared to the muscle weakness seen in X-linked hypophosphatemia (XLH)^30^ or tumor-induced osteomalacia (TIO)^31^. FGF23, which is low in HHRH, but elevated in XLH and TIO, was shown to induce left ventricular hypertrophy via activation of the calcineurin-NFAT signaling pathway^32^, but whether FGF23 impacts skeletal muscle function is unclear^33^. Initial clinical observations from the use of a novel FGF23 neutralizing therapy^34,35^ in XLH have shown marked improvement in fatigue, muscle weakness and activity, suggesting that this therapy offers an advantage over standard therapy (oral Pi and calcitriol). Likewise, the *Hyp* mouse model for XLH has reduced running activity and grip strength, and anti-FGF23 antibodies restore normophosphatemia, endurance and grip strength^36^. Although no short-term effect of FGF23 on muscle function was reported in *ex vivo* settings^37^, chronic exposure to this hormone may be negative for muscle strength as shown in *Dmp1* null mice^38^, which like Hyp mice have FGF23-dependent hypophosphatemia. In addition, in vitamin D-deficient individuals, cholecalciferol therapy was shown to improve mitochondrial activity and restore muscle function^39,40^. High levels of PTH have been shown to cause myopathy in mice independent of blood Pi, but the mechanism is not known^41,42^.

To determine the function of *Pit1* and *Pit2* in skeletal muscle independent of hypophosphatemia and the homeostatic endocrine changes resulting from hypophosphatemia, we generated a series of conditional knockout mice lacking one or two copies of *Slc20a1* and *Slc20a2* (*smPit1*^−/−^; *smPit2*^−/−^ mice), using the postnatally expressed *human skeletal actin (HSA)*-cre. These mice show a gene-dose dependent reduction in running activity resembling impaired endurance seen with hypophosphatemia, while interestingly grip strength in these mice is preserved. Furthermore, simultaneous deletion of both transporters in skeletal muscle in *smPit1*^−/−^; *smPit2*^−/−^ mice is lethal postnatally due to skeletal muscle hypoplasia/atrophy with myofiber degeneration, which appears to be caused by energy-stress induced myofiber necrosis.

## Results

### Generation of skeletal muscle-specific Pit1/2 double knockout mice

We used semi-quantitative RT-PCR to determine that *Pit1* and *Pit2* are the dominant Pi importers expressed in skeletal muscle (Fig. S1A). In addition to these two Pi importers, the putative Pi exporter *Xenotropic And Polytropic Retrovirus Receptor (Xpr1)* and the mitochondrial Pi importer *PIC/Slc25a3* are expressed at high levels. When compared to *Pit1*, *Pit2* mRNA levels are one order of magnitude higher in most tissues when estimated using this approach (Fig. S1B), which was verified with multiple primer sets.

**Figure 1:**
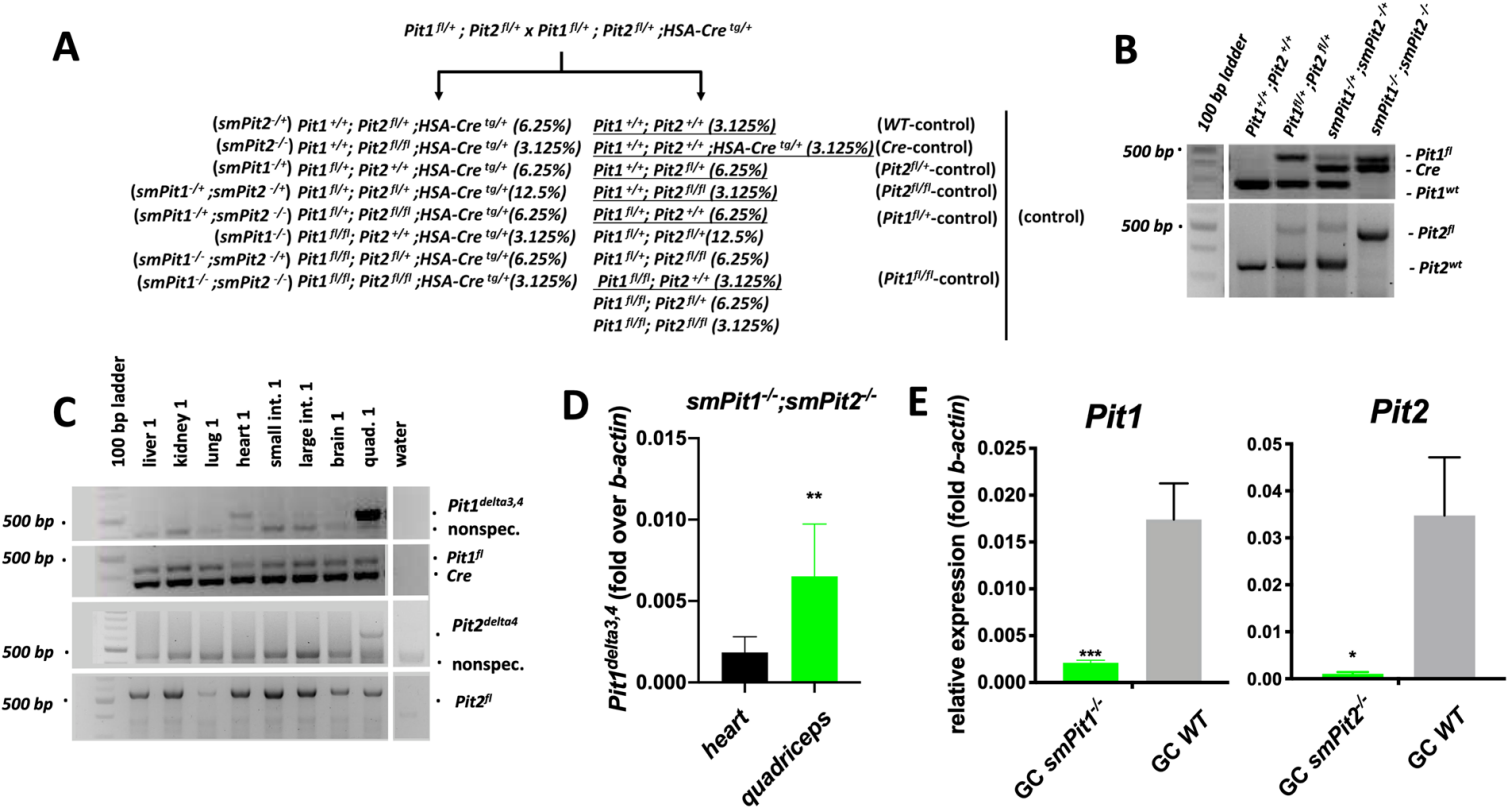
Generation of skeletal muscle-specific *smPit1*^−/−^*; smPit2*^−/−^ double knockout mice. (**A)** Mating scheme used to target ablation of *Pit1 and Pit2* in postnatal skeletal muscle with the human skeletal actin (HSA) promoter *Cre*. (**B)** Genotyping PCR for *Pit1*^*fl*^, *HSA-Cre*, and *Pit1*^*wt*^. (**C**) Genotyping PCR for the recombinant *Pit1*^*delta3,4*^ and *Pit2*^*delta4*^ in various tissues of a *smPit1*^−/−^*; smPit2*^−/−^ mouse at P10. For raw images see supplement. (**D**) Semi-quantitative qenomic PCR showed 20% reduced expression of the recombinant *Pit1*^*delta3,4*^ in heart when compared to quadriceps. For raw images see supplement. (**E**) Semi-quantitative RT-PCR showed reduced expression of *Pit1* and *Pit2* in quadriceps muscle of *smPit1*^−/−^ *and smPit2*^−/−^ mice at P80, respectively. Means ± SEM, n = 5–8, ^***^p = 0.0002, ^**^p = 0.002, ^*^p = 0.03 vs. WT.

To avoid the embryonic lethality observed in *Pit1* knockout animals^15^ and to avoid possible systemic changes of Pi homeostasis due to global ablation of *Pit2*^16^ we employed a conditional approach using cre-recombinase expressed under the human skeletal muscle actin promotor postnatally^43^. Mutant and WT littermates were generated from a single mating strategy depicted in (Fig. 1A). We confirmed inheritance of the floxed-*Pit1, -Pit2* and *Cre* alleles by PCR from genomic DNA prepared from tail clippings (Fig. 1B). Recombination resulting in excision of exons 3 and 4 of *Pit1* and of exon 4 of *Pit2* in quadriceps, but not in kidney, gut, brain, lung and liver was confirmed by genomic PCR (Fig. 1C) and consistent with previously reported expression of HSA-Cre using *Rosa26*^*tomato*^ reporter mice^44^. Low level recombination of *Pit1*^*fl/fl*^ was detected in heart (Fig. 1D and Fig. S1C), but *Pit1* and *Pit2* transcript levels were significantly decreased only in the skeletal muscle of *smPit1*^−/−^; *smPit2*^−/−^ mice (Fig. 1E), whereas mRNA levels of both transporters in heart were indistinguishable from WT (Fig. S1D).

### *smPit1*^−/−^; *smPit2*^−/−^ mice develop a severe myopathy and die before two weeks of life

**Figure 2:**
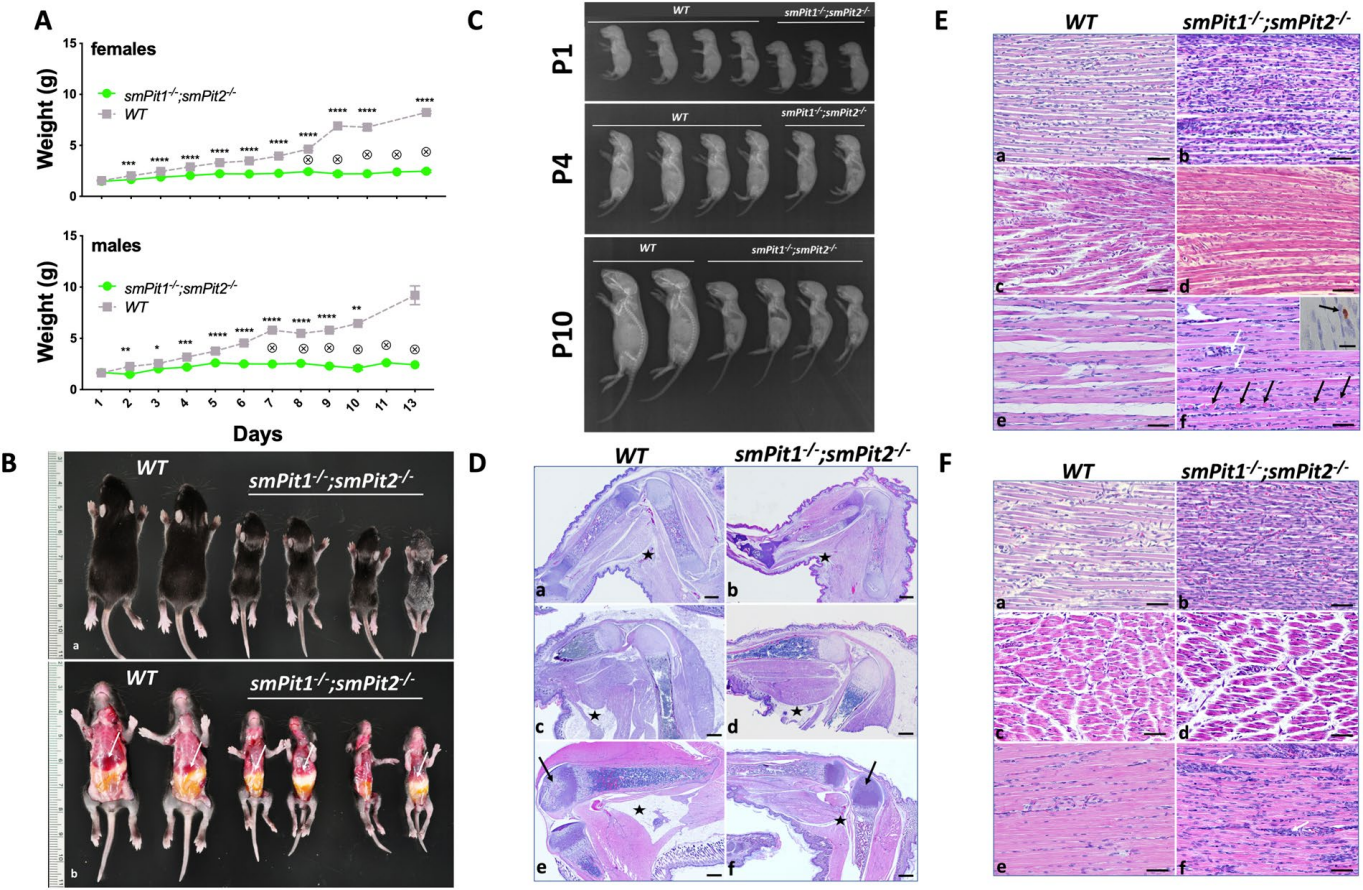
s*mPit1*^−/−^*; smPit2*^−/−^ mice die before two weeks of life due to starvation. (**A**) Growth retardation in *smPit1*^−/−^*; smPit2*^−/−^ mice was evident by P2. No difference was observed attributable to presence of *HSA-Cre, Pit1*^*fl*^ *or Pit2*^*fl*^ (See also Fig. S6B). Means ± SEM, n = indicated, ****p < 0.00002, ***p = 0.0002, **p = 0.002, *p = 0.03 vs. WT. (**B**) Gross phenotype (P10) shows that *smPit1*^−/−^*; smPit2*^−/−^ mice were much smaller but retain normal proportions, their stomachs were variably filled with milk, and their livers were atrophic (white arrow). (**C**) Radiographs, at P1, P4 and P10. Smaller size was evident in *smPit1*^−/−^*; smPit2*^−/−^ mice at P1, with reduced growth rate evident by P4, and markedly so by P10. Generalized retardation of mineralization of both axial and appendicular skeleton was noted in mutant animals. *smPit1*^−/−^*; smPit2*^−/−^ mice were proportionately comparable to WT littermates, indicating generalized delay in growth. (**D**) Hind limb bone, muscle and fat development at P1, P4 and P10. ***a, c, e***: WT; ***b, d, f***: *smPit1*^−/−^*; smPit2*^−/−^*. At P1, smPit1*^−/−^*; smPit2*^−/−^ mice were slightly smaller (***b***), and both genotypes had comparable minimal fat stores (asterisk, popliteal subcutaneous fat pad). At P4 and P10, *smPit1*^−/−^*; smPit2*^−/−^ mice exhibited generalized delayed growth, with reduced accumulation of skeletal muscle mass. Delayed ossification of the distal femoral epiphysis at P10 is indicated (black arrows, e, ***f***). Subcutaneous white adipose tissue accumulated in both genotypes through P4, however by P10, this is almost entirely lost in *smPit1*^−/−^*; smPit2*^−/−^ mice (asterisk, popliteal subcutaneous fat pad, ***c-f***). H&E, bar = 500 µm. (**E)** Muscle phenotype, anterior tibialis muscle, at P1, P4 and P10. ***a, b****:* P1. Compared to WT (***a***), myofibers in *smPit1*^−/−^*; smPit2*^−/−^ mice (***b***) were poorly organized, thinner, and display indistinct and irregularly distributed myofiber nuclei with increased overall cellularity. ***c, d****:* P4. Myofibers in *smPit1*^−/−^*; smPit2*^−/−^ mice (***d***) were slightly thinner than in WT (***c***) and had less robust but structurally comparable sarcomeric striation by light microscopy. ***e, f****:* P10. Compared to WT (***e***), myofibers in *smPit1*^−/−^*; smPit2*^−/−^ mice (***f***) were thinner, with abundant interstitial cellularity (white arrows), and frank myofiber disintegration (black arrows). Sarcomeric striation was retained but was less robust. Cleaved caspase-3 cytoplasmic immunostaining was noted in mutant muscle (inset). H&E, bar = 50 µm (***a–f***), 20 µm (Inset, ***f***) (**F)** Muscle phenotype, quadriceps femoris muscle, at P1, P4 and P10. ***a, b****:* P1. Compared to WT (***a***), myofibers in the mutant (***b***) were thinner with increased overall cellularity***. c, d***: P4. Myofibers in *smPit1*^−/−^*; smPit2*^−/−^ mice (***d***) were thinner than in WT (***c***), with less evident sarcomeric striation by light microscopy. Interstitial regions are expanded in mutant muscle. ***e, f****:* P10. Compared to WT (***e***), myofibers in *smPit1*^−/−^*; smPit2*^−/−^ mice (***f***) were thinner, with scattered aggregates of increased interstitial cellularity (white arrows). H&E, bar = 50 µm.

**Figure 3:**
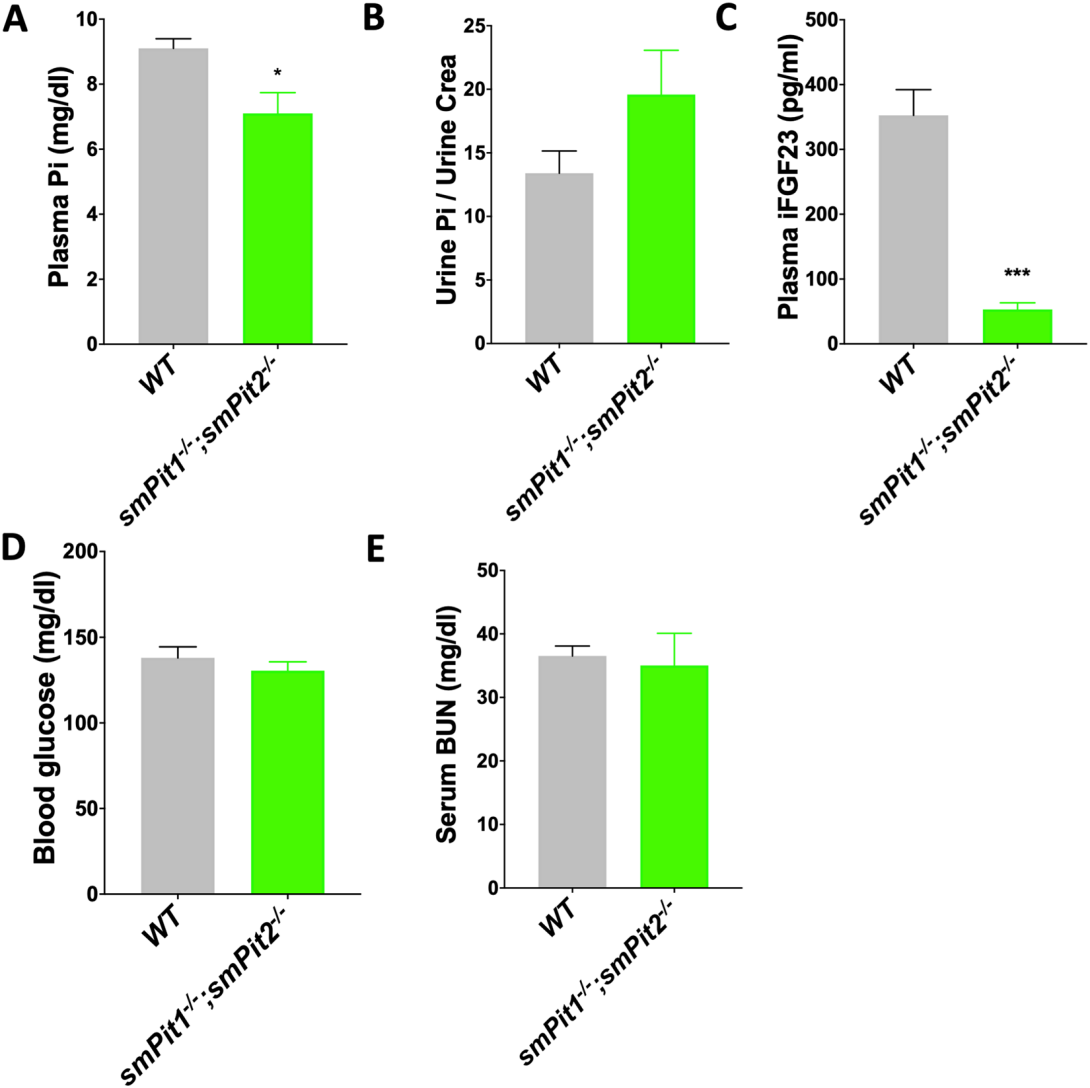
Skeletal muscle ablation of *Pit1 and Pit2* results in a mild renal Pi leak at P10. P10 *smPit1*^*−/−*^*; smPit2*^−/−^ mice were hypophosphatemic (**A**) and hyperphosphaturic (**B**) in an FGF23-independent fashion (**C**). Blood glucose (**D**) and BUN were within normal limits (**E**). Means ± SEM, n = 5–10, ^***^p = 0.0002, ^**^p = 0.002, ^*^p = 0.03 vs. WT.

Life birth frequency was close to expected based on Mendelian inheritance with occurrence of a few stillbirths as reflected in the dead/ungenotyped/missing pup number in (Table S3). Growth retardation of *smPit1*^−/−^; *smPit2*^−/−^ mice was evident by P2 (Fig. 2A,B), accompanied by low radiographic bone density (Fig. 2C) and by P10 they had severely impaired mobility (Video V1), inconsistent gastric filling (Fig. 2B) and depleted white adipose stores (Fig. S2D), suggesting that competition for food became progressively challenging for mutants. As a result, all *smPit1*^−/−^*; smPit2*^−/−^ mice died due to hyponutrition by P13. Failure-to-thrive developed likely secondary to myopathy, since myopathy preceded weight loss, was evident at birth and progressed to marked atrophy and myofiber degeneration by P10 (Fig. 2D–F, Fig S2A,B). At P1–4, compared to the WT animal, myofibers in *smPit1*^−/−^*; smPit2*^−/−^ mice were poorly organized, thinner and displayed indistinct and irregularly distributed myofiber nuclei with increased overall cellularity. Less robust but structurally comparable sarcomere striation was evident in *smPit1*^−/−^*; smPit2*^−/−^ mice by light microscopy. At P10, myofiber disintegration was evident, and fibers contained multiple subsarcolemmal nuclei indicative of regenerative attempts. Cleaved caspase-3 cytoplasmic immunostaining (Fig. 2E, inset) was noted in mutant muscle but absent in WT muscle. The diaphragm (Fig. S2B) was likewise affected, while cardiac muscle was histologically normal (Fig. S2E). Skeletal and organ development was qualitatively comparable to WT littermates but quantitatively delayed (Fig. 2D, Fig. S2E). Growth retardation and hyponutrition were supported by histologic evidence of overall smaller size of every structure, atrophic intestinal villi, reduced subcutaneous adipose stores (Fig. S2D, E), and delayed appearance of ossification centers in various bones (Fig. S2C). *smPit1*^−/−^*; smPit2*^−/−^ mice were hypophosphatemic and showed a trend toward hyperphosphaturia at P10 (Fig. 3A, B) along with appropriately suppressed intact FGF23 levels (Fig. 3C). Despite failure to thrive, mutant animals were normoglycemic (Fig. 3D). Absolute serum BUN levels were comparable in *smPit1*^−/−^*; smPit2*^−/−^ and WT mice (Fig. 3E) arguing against significantly impaired renal function of *smPit1*^−/−^*; smPit2*^−/−^ mice.

### Energy-stress in *smPit1*^−/−^*; smPit2*^−/−^ skeletal muscle leads to myofiber necrosis

**Figure 4:**
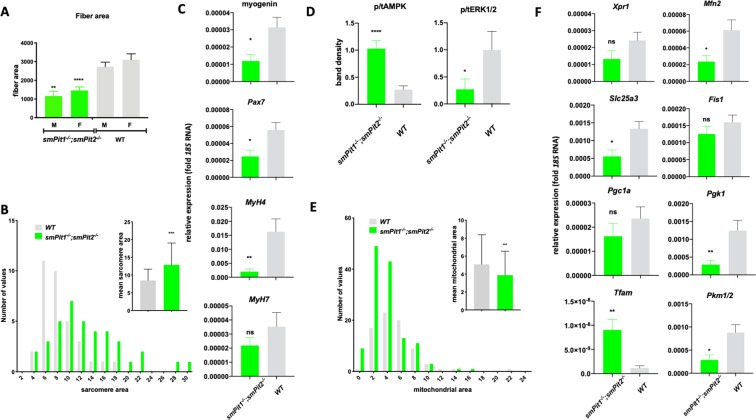
Energy stress results in reduced muscle fiber and increased myofibril (sarcomere) area in P10 *smPit*^−/−^*; smPit/2*^−/−^ mice. (**A)** *smPit1*^−/−^*; smPit2*^−/−^ mice showed a reduction of quadriceps fiber area at P10 in paraffin sections stained with H&E. **(B**) *smPit1*^−/−^*; smPit2*^−/−^ mice showed an increased gastrocnemius myofibril (sarcomere) area at P10 in TEM**. A** and **B** show arbitrary units following ImageJ analysis of these sections on the y-axis. (**C)** Semi-quantitative RT-PCR showed reduced expression of myoblast markers *myogenin,* *Pax7* and fast twitch *Myh4* myosin in P10 quadriceps muscle of *smPit1*^−/−^*; smPit2*^−/−^ mice **(D**) Densitometric analysis of an immunoblot (Fig. S4A) showing increased pAMPK/tAMPK ratio and reduced pERK1/2/tERK1/2 ratio in quadriceps of four different *smPit1*^−/−^*; smPit2*^−/−^ mice at P10. (**E**) Mitochondrial area and size distribution in TEM of GC at P10**. (F**) Semi-quantitative RT-PCR to determine gene expression of *Xpr1 and PiC* transcription factors and metabolic markers in quadriceps muscle of *smPit1*^−/−^*; smPit2*^−/−^ mice at P10. Shown are means ± SEM, n = 3–10, ^****^p < 0.00002, ^***^p = 0.0002, ^**^p = 0.002, ^*^p = 0.03 vs. WT.

Histological changes observed in all limb, epaxial, and diaphragm muscles from *smPit1*^−/−^*; smPit2*^*−/−*^ mice include reduction of fiber area starting at P1, which worsened by P10 (Fig. 4A). Overt fiber necrosis seen at P10 under light microscopy were accompanied by a shift to thicker myofibrils (sarcomeres) seen on TEM (Fig. 4B), by reduced myoblast marker expression, and by increased type II *Myh4* fast twitch muscle myosin expression in quadriceps muscle (Fig. 4C). There was no evidence for diffuse rhabdomyolyis upon Evans Blue exclusion staining (Fig. S3A) or endoplasmic reticulum (ER) stress based on normal *Xbp1* splicing (Fig. S3B) and *Chop* expression (Fig. S3C) and normal appearance of the rough ER in TEM (Fig. S3G, arrows), which was recently reported in mice lacking *Pit1* in growth plate chondrocytes^45^. There were likewise no signs for proteasomal activation based on normal *Fbxo32* expression (Fig. S3D) or for autophagy based on absence of autophagosomes in TEM (Fig. S3G and Fig. S4C,D). Conversely, ERK1/2 activation was reduced in P12 quadriceps muscle obtained from *smPit1*^−/−^*; smPit2*^−/−^ mice, and AMPK phosphorylation was increased (Fig. 4D), consistent with increased energy-stress and reduced muscle ATP production previously described by us^6^ in the setting of hypophosphatemia. Evaluation by TEM showed reduced muscle mitochondrial area (Fig. 4E), distended mitochondrial cristae and irregular mitochondrial matrix (Fig. S4C, D). Furthermore, a small, non-significant decrease of mitochondrial DNA was seen by semi-quantitative genomic PCR (Fig. S4B). Semi-quantitative RT-PCR analysis (Fig. 4F), furthermore, showed decreased expression of the Pi transporter *Slc25a3*, the mitochondrial *mitofusin 2*, the glycolytic enzymes *phosphoglycerate kinase* (*Pgk1*) and *pyruvate kinase* (*Pkm1/2*), while putative Pi exporter *Xpr1*, *peroxisome proliferator-activated receptor gamma coactivator 1 alpha* (*Pgc1a) and Fis1* expression was unchanged and *mitochondrial transcription factor A (Tfam)* expression was increased.

### Reduced oxygen consumption rate (OCR) in the absence of Pi is consistent with energy-stress in C2C12 myoblasts

**Figure 5:**
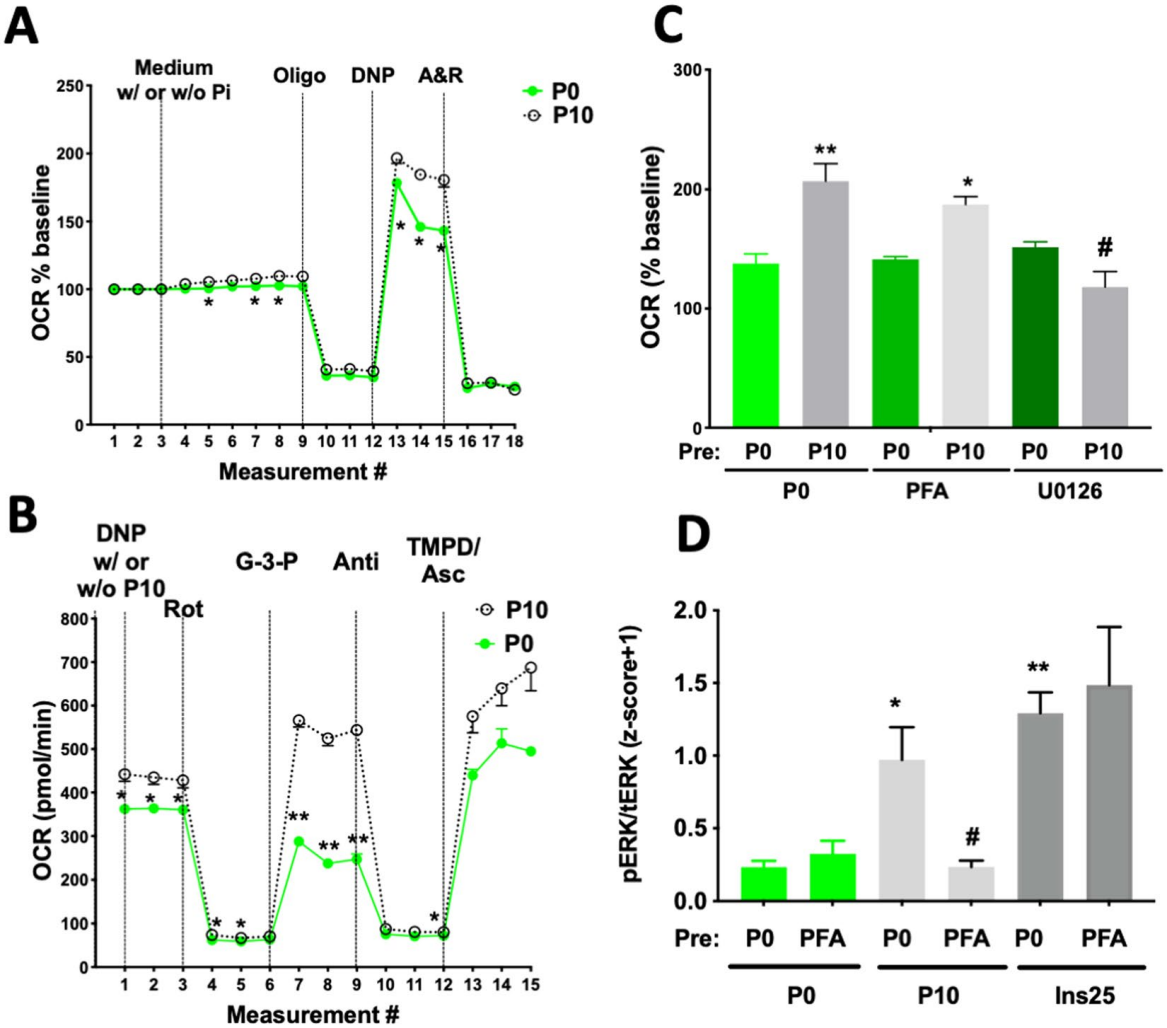
Respiratory chain activity of C2C12 cells. (**A**) Individual tracing of a coupling assay using intact C2C12 cells and a Seahorse XFe24 analyzer showed reduced oxygen consumption rate (OCR) in cells deprived of Pi (P0) when compared to cells exposed to 10 mM Pi (P10). (**B**) Individual tracing of a mitochondrial electron-flow assay in permeabilized C2C12 cells in the absence (P0) and in the presence of 10 mM Pi (P10). To stimulate individual complexes, 1 nM Plasma Membrane Permeabilizer (Seahorse) and 100 nM dinitrophenol (DNP) was used along with 10 mM pyruvate/1 mM malate (complex C-I), 1 uM rotenone/10 mM glycerol-3-phosphate (complex C-III) or 10 mM ascorbate/100 nM N,N,N',N'-tetramethyl-1,4-phenylenediamine (TMPD) (complex C-IV), respectively. **(C**) Summary of several coupling assays as described in **A**. Pre-treatment with phosphonoformic acid (5 mM PFA), an inhibitor of Pit1, and U0126 (30 uM), an inhibitor of MEK1/2, the kinase upstream of ERK1/2, attenuated the Pi-dependent increase in maximal respiration (**D**) Summary of several immunoblots of C2C12 lysates following pre-treatment with 5 mM PFA which inhibited stimulation of pERK1/2 by 10 mM Pi (P10), but not by 25 uM human insulin (Ins25). Densitometric results were averaged following z-score transformation of the of individual blots. Shown are means ± SEM, n = 3–5, ^****^p < 0.00002, ^***^p = 0.0002, ^**^p = 0.002, ^*^p = 0.03 vs. pre-P0/P0. ^#^p = 0.03 vs^.^ pre-P0/P10 (**C**) and vs. pre-P0/P10 (**D**).

We next modeled Pi effects using a Seahorse Analyzer in murine C2C12 myoblast cells. Consistent with our observation of energy-stress following *Pit1* and *Pit2* transporter ablation *in vivo*, acute response and maximum respiration in C2C12 myoblasts were reduced in the absence of Pi (Fig. 5A). Using permeabilized C2C12 myoblast and selective substrate/inhibitor conditions we were able to test individual mitochondrial complexes. OCR increased with addition of Pi to the mitochondrial stimulation buffer (Fig. 5B). The largest Pi effect was seen when testing complex III dependent respiration following addition of glucose-3-phosphate (G3P, Fig. 5B). Stimulation of OCR by Pi requires activation of ERK1/2, since it can be blocked by pretreatment with the MEK inhibitor UO126 (Fig. 5C). Activation of ERK1/2 by Pi was previously reported for many other cell lines^11^ and is blocked in C2C12 cells by ablation of *Pit1* and *Pit2* (Fig. S5A,B), or by pre-treatment with phosphonoformic acid (PFA), an inhibitor of Pi transport^46^, while PFA does not block insulin-mediated activation of ERK1/2 (Fig. 5D).

### Loss of three alleles of *Pit1 and Pit2* in skeletal muscle impairs endurance but not grip strength

**Figure 6:**
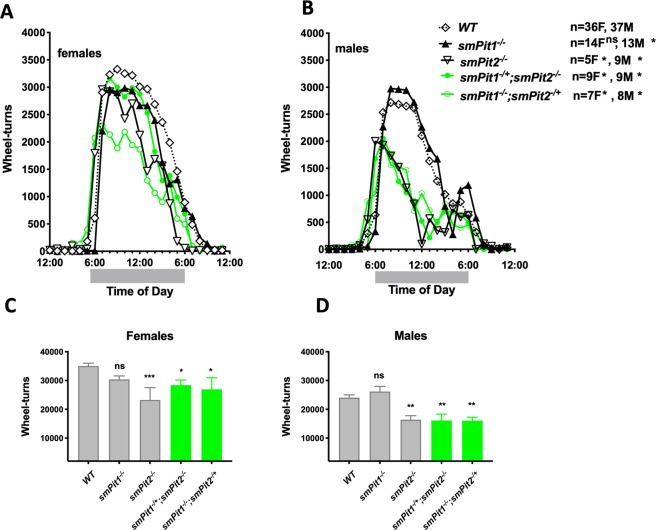
Loss of three alleles of skeletal muscle *Pit1 and Pit2* impairs muscle function. Voluntary hourly wheel-running activity on day 6 of a daily wheel-running study of P80 females (**A, C**) and males (**B, D)** was reduced in a gene-dose dependent fashion. Means ± SEM, n = 5–36, ^***^p = 0.0002, ^**^p = 0.002, ^*^p = 0.03 vs. WT.

Different from *smPit1*^−/−^*; smPit2*^−/−^ mice, single and three allele conditional mutants were fertile, thrived normally and had body weights comparable to wildtype animals (Fig. S6D). However, voluntary wheel running (VWR) activity was reduced at P80 for *smPit2*^−/−^ and *smPit1*^−/−^*; smPit2*^−/+^ females and for *smPit2*^*−/−*^*, smPit1*^−/+^*; smPit2*^−/−^, and *smPit1*^−/−^*; smPit2*^−/+^ males (Fig. 6A–D). The animals took more frequent breaks, which was particularly noticeable around midnight in males (Fig. 6A). VWR activity tests endurance of predominantly slow type I muscles of the lower extremities. Interestingly, a small, but significant improvement of VWR activity was seen in *smPit1*^−/−^ males. No difference was observed in grip strength (GS) between Pi transporter mutant mice and littermate controls (Fig. S6C). GS tests impact of Pi transporter ablation on fast twitch type II forearm muscles, increased significantly with age and males were stronger than females (Fig. S6A). GS was unchanged even when corrected separately for WT1 and WT2 of each breeding cohorts. No weight difference was detected for the *HSA-Cre, Pit1*^*fl*^ *or Pit2*^*fl*^ alleles (Fig. S6B,D).

## Discussion

We here show that *smPit1*^−/−^*; smPit2*^−/−^ mice develop a severe myopathy and die soon after birth. This at first was unexpected since no apparent skeletal muscle phenotype has thus far been described in the respective single knockout mice^15–19^ and suggests that *Pit1* and *Pit2* compensate for each other in skeletal muscle. Our findings in *smPit1*^−/−^*; smPit2*^−/−^ mice therefore, for the first time, prove that *Pit1 and Pit2* are required for normal myocyte function and survival. Furthermore, subtle deficiencies in running wheel endurance were observed in *smPit1*^−/−^ *and smPit2*^−/−^ mice, which might have previously been overlooked in the single global knockout mice.

Pathological evaluation showed features of skeletal myopathy at birth, when *smPit1*^−/−^*; smPit2*^−/−^ mice still appeared otherwise normal compared to their WT littermates, suggesting that this myopathy causes the subsequent failure-to-thrive phenotype of these mice, possibly because myopathy reduces their ability to suckle. As a result *smPit1*^−/−^*; smPit2*^−/−^ mice died by P13, to which respiratory failure may contribute since their diaphragms showed similar myopathic changes. Conversely, heart failure was likely not an important contributing factor to death in these mice, since *HSA-cre* did not reduce transporter mRNA levels in this organ. Furthermore, cardiac muscle appeared histologically normal at the time of death of *smPit1*^*−/−*^*; smPit2*^−/−^ mice when compared to WT littermates.

**Figure 7:**
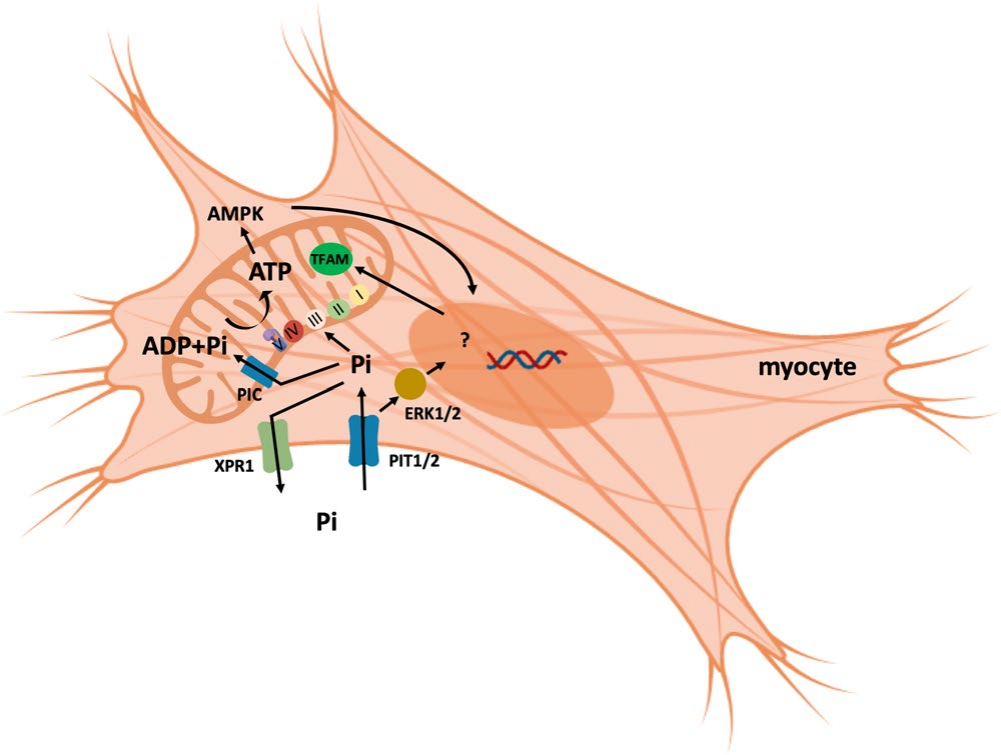
Metabolic Pi-signaling in myocytes. We here provide evidence for an important function of *Pit1* and *Pit2* to increase intracellular Pi as a substrate for mitochondrial ATP synthesis and/or as a signal to stimulate mitochondrial respiration. *Pit1* and *Pit2* may also serve as “transceptors” upon binding of blood Pi to activate ERK1/2 in a transport-independent fashion to stimulate myocyte proliferation and differentiation and mitochondrial respiration.

Previous studies suggested that decreased ATP^3,4^ and phosphodiesters^5^ may in part explain the muscle weakness seen in hypophosphatemia. We recently reported that hypophosphatemic myopathy goes along with reduced ATP flux (V_ATP_) and IC Pi in an individual with hereditary hypophosphatemic rickets with hypercalciuria (HHRH) and in the sodium-Pi co-transporter *Npt2a* null mouse^6^. Consistent with reduced ATP production we observed activation of the energy sensor AMPK, increased *Tfam* expression and abnormal mitochondrial morphology in skeletal muscle of *smPit1*^−/−^*; smPit2*^−/−^ mice, which may be a result of energy stress^47^ and provide an explanation for the myodegeneration and muscle necrosis seen in these mice (Fig. 7). pAMPK activates *PGC1a* which induces nuclear transcription of *Tfam* and along with p53 translocates to the mitochondria where PGC1a and p53 interact with *Tfam* to induce mitochondrial biogenesis, to improve respiratory chain function and to decrease *Hif1a* activity as reviewed in^48^. Mice lacking skeletal muscle *Tfam* exhibit exercise intolerance and myopathy^49^ resembling the one observed in *smPit1*^−/−^*; smPit2*^−/−^ mice.

In addition to its function as a substrate for ATP production, Pi activates ERK1/2 in myocytes like in many other cells^22^ (Fig. 7). Consequently, ERK1/2 phosphorylation was reduced in quadriceps muscle from *smPit1*^−/−^*; smPit2*^*−/−*^ mice. Our initial observations in murine C2C12 myoblasts suggest that both intracellular and membrane signaling pathways appear to be required for stimulation of mitochondrial respiration by Pi. Furthermore, expression of the muscle stem cell (MSCs) markers *Pax7* and *myogenin*, which are known to be regulated by ERK1/2^50^, was suppressed, and may provide a mechanism, whereby muscle stem cells (MSCs) and regeneration is impaired by ablation of *Pit1* and *Pit2*. However, postnatal worsening of muscle degeneration and presence of multiple subsarcolemmal nuclei indicative of regenerative attempts despite low levels of these two transcription factors suggests that *Pit1* and *Pit2* are primarily important for muscle fiber survival.

Muscle-specific loss of ERK1/2 was also found to cause loss of neuromuscular integrity in mice^28^, which may contribute to the myopathy in *smPit1*^−/−^*; smPit2*^−/−^ mice. Finally, *Pit1* promotes other transport-independent processes such as proliferation^20^ and TNF-induced apoptosis in HeLa cells^21^ and insulin resistance in hepatocytes^51^. These pathways may be relevant in skeletal muscle as well and may be of interest for further investigation.

Compensation for loss of *Pit1* and *Pit2* in skeletal muscle may occur on the fiber, tissue and organism level and aim at reducing Pi consumption and keeping IC Pi stable. On the fiber level we observed down-regulation of the mitochondrial Pi importer *Slc25A3 (PIC), increased Tfam*, decreased *Mfn2* levels and abnormal mitochondrial matrix, which may be aimed at improving mitochondrial function and resemble adaptive changes during exercise^52^.

Mutations in human *SLC25A3/PIC* cause a severe myopathy affecting skeletal and cardiac muscle in humans^53,54^ and in mice^55^. Thus, down-regulation of this transporter may preserve cytosolic Pi, but since mitochondrial uptake of Pi is important for ATP synthesis, this in turn could further worsen muscular energy stress.

On the tissue level we observed a shift to a slow twitch fiber type, which may reflect a compensatory process in response to muscle energy-stress in these fibers which have greater levels of *Pit1* and Pi than fast twitch fibers^13,14^. AMPK plays an important role in regulating muscle mass and regeneration and must be kept within relatively tight boundaries (not too high or too low) for optimal muscle regeneration^47^. AMPK subunit α1 stimulates anabolism and regulates satellite cell dynamics during regeneration, whereas AMPK subunit α2 regulates muscle degradation during atrophy. Energy stress predominantly activates AMPK subunit α2 via liver kinase B1 (LKB1), while AMPK subunit α1 is only activated by highly‐intense or prolonged exercise, perhaps via Calcium/calmodulin‐dependent protein kinase (CamKK) action, or other AMPK kinases. AMPK subunit α2 stimulates catabolic processes by increasing Foxo3a, Atrogin‐1 and MuRF‐1 expression/activity and increasing autophagy, leading to muscle atrophy under certain circumstances, but has little effect on protein synthesis. AMPK subunit α1 impairs mTOR signaling, slows protein synthesis, and blocks hypertrophy. It will be of interest to determine, which AMPK isoforms contribute to the myopathy in *smPit1*^−/−^*; smPit2*^−/−^ mice and to see, whether expression patterns likewise resemble physiological adaptation to chronic endurance exercise^56,57^.

On the systemic level we found that *smPit1*^−/−^*; smPit2*^−/−^ mice have significantly lower blood Pi levels, which appears to be a result of renal Pi excretion independent of FGF23. Since *HSA-cre* is not expressed in the kidneys, we speculate that skeletal muscle of *smPit1*^−/−^*; smPit2*^−/−^ mice secretes a myocyte factor that increases renal excretion of Pi. Candidate muscle-derived factors include IGF-1, FGF-2, IL-15, irisin myostatin and L-BAIBA^33^, which will be subject of future investigation. Bone loss observed in these mice may be secondary to this renal Pi leak, due to direct action of one of the above myokines on bone metabolism, and/or due to failure to thrive of these mice.

Three allele mutants display reduced VWR activity which further supports the hypothesis that *Pit1* and *Pit2* are important predominantly for slow twitch fibers^4–6,58^ as already suggested by the possible shift from fast *Myh4* positive fibers to slow *Myh7* positive fibers in quadriceps muscle of *smPit1*^−/−^*; smPit2*^−/−^ mice. Furthermore, subtle deficiencies in running wheel endurance were also observed in the single conditional mutant *smPit1*^−/−^ *and smPit2*^−/−^ mice, which may have been previously missed during evaluation of the global knockouts for these genes^15–19^. Interestingly, GS is not affected in mice lacking two or three transporter alleles, which is different from observations in the *Hyp* mouse model for XLH that displays reduced GS along with reduced VWR activity, and which both are restored by treatment of *Hyp* mice with anti-FGF23 antibodies^36^. Likewise, *ex-vivo* tetanic strength was impaired in *Dmp1* null mice^38^, which like *Hyp* have elevated FGF23 levels. It is thus conceivable that FGF23 impacts skeletal muscle function^33^ similar to its effect on the cardiac muscle^32^. Our *Pit1* and *Pit2* three allele knockout mice may be a good model to separate effects of hypophosphatemia from effects caused by adaptive hormonal changes as a result of hypophosphatemia and improve our understanding of the pathophysiology of the myopathy seen in hypophosphatemic disorders such as HHRH or XLH^59^.

Our observations are currently limited by the early failure-to-thrive phenotype of the *smPit1*^−/−^*; smPit2*^−/−^ mice. To further evaluate the role of *Pit1* and *Pit2* for muscle function and survival in adult mice, we next plan to use an inducible muscle-specific Cre, for example *HSA-Cre*^*rtTA*^^60^. Also oral and parenteral Pi supplementation to normalize blood Pi in *smPit1*^−/−^*; smPit2*^−/−^ and three allele knockout mice could be attempted, however, placental Pi transport appears to isolate pups from dietary changes of Pi in the mother^61^ and thus such pharmacological rescue experiments are likely only feasible postnatally. Of interest will also be genetic rescue experiments using a cross of *smPit1*^−/−^*; smPit2*^−/−^ mice with transgenic mice^62^ or adenoviral constructs expressing WT and transport-deficient *[K71]hPIT1*, to test whether substrate-uptake is required to rescue the myopathy of *smPit1*^−/−^*; smPit2*^−/−^ mice. It may also be of interest to test whether ablation of *Xpr1* can rescue the myopathy. *Xpr1* showed a trend toward what may be interpreted as compensatory down regulation in Fig. 4F and was recently shown to be a widely expressed putative Pi exporter^63,64^, however, its functions in skeletal muscle are completely unknown. *Xpr1* contains an SPX motif, which binds inositol-pyrophosphate (5-InsP_7_), a compound shown in yeast to communicate IC Pi levels and to regulate the Pi starvation response^65^. Human mutations in *XPR1* cause primary familial brain calcification^63,64^. Kidney-specific deletion of *Xpr1* leads to a decrease in the NAD + /NADH ratio with reduced availability of NAD + for synthetic processes and development of Fanconi syndrome^66^. As a result, down-regulation of this transporter may preserve cellular Pi and reduce Pi consumption through synthetic processes.

In conclusion, we here show for the first time that *Pit1* and *Pit2* are essential for normal myofiber function and survival. Hypophosphatemic myopathy is currently treated with Pi supplementation^30,59^. However, Pi supplements are poorly tolerated and can cause intestinal symptoms, renal calcifications and secondary hyperparathyroidism^67^. Insights gained from *smPit1*^−/−^*; smPit2*^−/−^ and three allele knockout mice suggest, that optimizing AMPK and ERK1/2 signaling and modification of *XPR1* and *PIC/SLC25A3* activity may offer alternative strategies for the treatment of hypophosphatemic myopathy.

## Methods

### Mice and diets

*smPit1*^−/−^ *and smPit2*^−/−^ *mice*, which selectively lack the Pi transporters *Pit1(Slc20a1)* and *Pit2 (Slc20a2)*, respectively, in skeletal muscle were generated using *Pit1*^*fl/fl*^ mice obtained from Dr. Giachelli^68^, and *HSA-Cre* mice obtained from Jackson Laboratory (human skeletal actin-Cre, *B6.Cg-Tg(ACTA1-cre)*^*79Jme/J*^; Jackson stock No: 006149)^43^. Embryonic stem cell clone EPD0243_1_C06 with *loxP* sites flanking exon-4 of the *Slc20a2/Pit2* gene were purchased from the International Knockout Mouse Consortium (IKMC), European Conditional Mouse Mutagenesis Program (EUCOMM). Exon-4, which contains 89 bp, encodes for amino acids 144-172 and its Cre-mediated removal leads to a frame shift and an early stop codon 340 bp downstream. Conditional-ready *Pit2* mice were generated by morula aggregation in the Labortierkunde (LTK) of the University of Zurich and mated with *Flp-*deleter mice (63Flp3B6) purchased from the Jackson Laboratory to generate *Pit2*^*fl/fl*^ mice (Pastor-Arroyo et al, in preparation)^69^.

All mice were generated from a C57Bl/6 background and experimental mice were generated using the mating strategy of *Pit1*^*fl/*+^*; Pit2*^*fl/*+^ females x *Pit1*^*fl/*+^*; Pit2*^*fl/*+^*; HSA-Cre*^*tg/*+^ males and Cre-negative littermates were used as controls to minimize bias related to a specific genetic background. Mice were weaned at 3 weeks of age and allowed free access to water and standard chow (1.0% calcium, 0.7% Pi (0.3% bioaccessible Pi; TD.2018S; Harlan Teklad, Madison, WI, USA).

Genotyping at weaning was performed by PCR amplification of genomic DNA extracted from tail clippings using the HotSHOT method^70^ and amplified by polymerase chain reaction (PCR) as previously described^71^ using the primers shown in Table S1. Briefly, 75uL alkaline lysis buffer (25mN NaOH, 0.2 mM EDTA) was aliquoted to 1.5 mL tubes containing tissue. Lysates were incubated for 30 min at 95 °C, then neutralized with 75uL neutralization buffer (40 mM Tris-HCl). Genomic DNA was amplified by PCR to genotype mice and determine skeletal muscle-specific ablation of *Pit1*. Primer pairs used were: wild-type (*Pit1*^*wt*^) and Pit1-floxed (*Pit1*^*fl*^) (F) 819 and (R) 820; wild-type (*Pit1*^*wt*^) and delta allele (*Pit1*^*Δe3,4*^): (F) 819 and (R) 822; ACTA1-cre (*HSA-Cre*): (F) 823 and (R) 824^68^ and wild-type (*Pit2*^*wt*^), Pit2-floxed (*Pit2*^*fl*^) (F) 954 and (R) 955, and delta allele (*Pit2*^*Δe4*^) (F) 995 and (R) 996 (Table S1).

Animal research for this study was first approved on October 22, 2014 by the Yale Institutional Animal Care and Use Committee (protocol 2014–11635), was renewed on September 7, 2016, and is valid through Sept. 30, 2020. Yale University has an approved Animal Welfare Assurance (#A3230–01) on file with the NIH Office of Laboratory Animal Welfare. The Assurance was approved on May 5, 2015.

### Anatomic phenotyping

A total of 34 animals (Table S2) across 5 litters (P1, P4, P8 and P10) were examined grossly. These included 13 *smPit1*^−/−^*; smPit2*^−/−^ animals with wild-type animals comprising the balance. Animal histopathology was performed on 21 animals (indicated by histopathology numbers in Table S2). Of these, images from animals at P1, P4, and P10 were used to illustrate pathologic findings. Animals were evenly split by sex and genotype. All the methods for the study were carried out in accordance with relevant guidelines and according to the 2013 AVMA guidelines for the euthanasia of animals. Following gross photography, radiography and collection of blood for glucose measurement, soft and bony tissues from all animals were immersed in 10% (w/v) neutral buffered formalin or Bouin’s solution respectively for one week. Cassetted tissues were embedded in paraffin, sectioned at 5 μm, and mounted on glass slides. Sections were stained with hematoxylin and eosin (H&E), examined using a Zeiss Axioskop light microscope and imaged using a Zeiss AxioCam MrC camera.

### Transmission electron microscopy

For transmission electron microscopy a 1 mm^3^ block of the left kidney was fixed in 2.5% glutaraldehyde and 2% paraformaldehyde in 0.1 M Cacodylate buffer for 2 hrs., followed by post-fixation in 1% osmium liquid for 2 hours. Following dehydration using a series of ethanol concentrations (50% to 100%), tissue was embedded in epoxy resin, and polymerization was carried out at 60 °C for overnight as previously described^62^. After preparing a thin section (50 nm), the tissues were double stained with uranium and lead and observed using a Tecnai Biotwin (LaB6, 80 kV) (FEI, Thermo Fisher, Hillsboro, OR) at the Yale Center for Cellular and Molecular Imaging (YCCMI).

### Evan’s Blue staining

To evaluate muscle fiber integrity, 1% Evans blue dye dissolved in sterile saline was administered by intraperitoneal injection at a dose of 1% of body weight to WT and *smPit1*^−/−^*; smPit2*^−/−^ mice at age P10, followed by sacrifice after 24 hrs. and dissection of muscles that were snap-frozen in liquid nitrogen. Muscle cryosections of 10-μm thickness were cut at −21 °C on a Leica (CM3050) cryostat, dipped in cold acetone (−20 °C) for 1 min and then air-dried at room temperature (20–22 °C) (RT). Sections were dipped in xylene (Merck) and mounted with DPx (BDH, Poole, UK) and a glass coverslip. Evans blue was viewed by confocal fluorescent microscopy SP8 LIGHTNING confocal microscope (Leica, Buffalo Grove, IL, USA) at 620 nm excitation and 680 nm emission as described^72^.

### Radiology

Following euthanasia, animals were radiographed in dorsovental and lateral recumbancy using a Universal radiology unit and AGFA Small Animal Digital Radiography system (Agfa HealthCare, Mortsel, Belgium).

### Blood and urine parameters

Blood and spot urines were collected as previously described^71^. Glucose measurements as a measure of recent feeding were taken immediately after removal of the pups from the mother at euthanasia using a handheld glucometer (Accu-Chek, Roche, Switzerland). Creatinine levels were measured by HPLC/MS/MS commercially from serum samples collected at euthanasia (Antech, Fountain Valley, CA, USA). BUN was measured from serum samples using kit 0580 (Stanbio, Boerne, TX, USA). The COBAS Mira Plus automated chemistry analyzer (Roche Diagnostics, Pleasanton, CA, USA) was used to determine plasma and urine inorganic phosphorus. Urinary creatinine was measured by colorimetric assay (DICT-500; BioAssay Systems, Hayward, CA, USA). Concentrations of serum intact fibroblast growth factor (FGF)-23 protein were determined using mouse FGF-23 ELISA assays (60–6500; Immutopics, San Clemente, CA, USA).

### Muscle physiology

Forelimb grip strength was measured with the Mouse Grip Strength Meter (1027SM; Columbus Instruments, Columbus, OH, USA) at ages 30, 60, and 120 days. The average grip strength was calculated from three trials conducted in series. Voluntary wheel-running (VWR) was assessed using home cages equipped with running wheels (0297–8; Columbus Instruments) using experimental procedures previously described by other groups at the Yale School of Medicine^73,74^. Briefly, mice were individually housed for one week and maintained on a 12 h light/dark cycle. Food and water were provided *ad libitum*. The number of wheel-turns was continuously recorded by ClockLab software (Actimetrics, Wilmette, IL, USA). Data files were exported to MATLAB and converted to Excel spreadsheets using Clocklab Analysis (Actimetrics, Wilmette, IL, USA).

### Genomic DNA and RNA extraction and allele-specific PCR from various mouse tissues and C2C12 cells

Gastrocnemius and quadriceps muscle tissue were dissected immediately following sacrifice, snap-frozen (by compression between aluminum clamps pre-chilled in liquid nitrogen), and stored at −70 ^o^C. Genomic DNA was extracted from tissues using the HotSHOT method^70^ described above. Total RNA from tissues was extracted using TRIzol (15596026; Invitrogen, Carlsbad, CA, USA). Total RNA from C2C12 cells was prepared using RNeasy columns (Qiagen, Valencia, CA). 0.5–2 ug total RNA were reverse-transcribed using the Omniscript RT Kit (205113; Qiagen, Valencia, CA, USA). Semi-quantitative genomic or reverse transcription (RT) PCR was performed with SYBR Green (204143; Quantitect, Qiagen) using the ABI-Step One Plus Cycler (Fisher, Life Technologies, Waltham, MA, USA) and analyzed using the 2-ΔΔCt method. Primer sequences were designed using PrimerBank^75^ and are shown Table S1. *b-actin* and *18 S RNA* gave comparable results as internal controls (see Fig. S3E,F) and ABI Cycler efficiency measures were all acceptable.

### Protein extraction and immunoblot analysis from various mouse tissues and C2C12 cells

Skeletal muscle tissue was homogenized as previously described^62^ in ice-cold RIPA buffer (50 mM Tris HCL (pH 6.8), 150 mM NaCl, 1% Triton X-100, 0.5% sodium deoxycholate, 0.1% sodium dodecyl sulfate, Protease inhibitor without EDTA (Roche, Mannheim, Germany), 0.4 mM Na-orthovanadate, 50 mM beta-GP, 5 mM NaF). Lysates were incubated on an orbital shaker for 2 hours then centrifuged for 20 min at 12,000 g at 4 °C. Protein amount was measured using a BCA assay (Thermo Fisher Scientific, Waltham, MA, USA). 50 ug of protein were run on a PAGE gel electrophoresis on 10% Tris-HCl/Glycine SDS-polyacrylamide (456–1034, BioRad, CA), electro-transfer to PVDF membranes (162–0218, BioRad, CA, USA) and hybridized with anti-phospho-ERK1/2 (9106, Cell Signaling Technology, MA, or 4730, Cell Signaling Technology, MA, USA) or anti-total-ERK1/2 antibody (4695, Cell Signaling Technology, MA, USA) 1:1000 in phosphate buffered saline containing 0.1% Tween 20 (PBST) and 5% non-fat dry milk at 4 °C overnight, or hybridized with pAMPK rabbit antibody (2535, Cell Signaling Technology, MA, USA, the antibody detects both α1 and α2 isoforms of the catalytic subunit, but does not detect the regulatory β or γ subunits) 1:1000 or tAMPK rabbit antibody (2603, Cell Signaling Technology, MA, USA) 1:1000 in tris buffered saline containing 0.1% Tween 20 (TBST) and 5% BSA (A7888, Sigma, MA, USA) at 4 °C overnight, and detected with horseradish-peroxidase conjugated anti-rabbit IgG (7074 S, Cell Signaling Technology, MA) in PBST + 5% non-fat dry milk at room temperature for 60 min using chemiluminescence/autoradiography (Supersignal 34580, Thermo Fisher Scientific, Waltham, MA, USA).

For C2C12 cells following pretreatment and stimulation the 48-well plate was placed on ice and the culture medium was aspirated. Cells were then lysed with 50 ul lysis buffer (62.5 mM Tris HCL (pH 6.8), 1% SDS, 1 mM EDTA, 1 mM EGTA, 0.05 TiU/ml aprotonin, 1 M PMSF, 100 mM Na-orthovanadate, 0.8% SDS, 3.2% glycerol, 2% beta mercaptoethanol, 0.0015% bromophenolblue). 15 ul cell lysates were then separated, electro-transferred to PVDF membranes and hybridized with anti-phospho-ERK1/2 (9106, Cell Signaling Technology, MA, USA) or (4730, Cell Signaling Technology, MA, USA) or total-ERK1/2 antibody (4695, Cell Signaling Technology, MA, USA) in phosphate buffered saline containing 0.1% Tween 20 (PBST) and 5% non-fat dry milk at 4 °C overnight and detected as described for skeletal muscle lysates above. Following densitometric analysis of the autoradiograms, phospho/total ERK ratios were converted into z-scores to permit statistical evaluation of pooled Western blot experiments.

### C2C12 myoblast cell culture

C2C12 myoblasts were cultured at 37 ^o^C under 5% CO_2_ for up to 8 weeks in high glucose DMEM (pH 7.4; 11965–092; Gibco, Gaitherburg, MD, USA) supplemented with 10% fetal bovine serum (F4135; Sigma, St. Louis, MO, USA). Medium was changed every 3–4 days and cells were split (1:10) once a week with 0.25% trypsin/EDTA. Pi-free DMEM (pH 7.4; 11971–025; Gibco, Gaitherburg, MD, USA) was substituted for complete DMEM when indicated. Human insulin (91077 C, Sigma, St. Louis, MO, USA) was used at a final concentration of 25 ug/ml. The MAP kinase inhibitor UO126 (19–147; Sigma, St. Louis, MO, USA) and the PI-3 kinase inhibitor Ly294002 (L9908; Sigma, St. Louis, MO, USA) were suspended in DMSO and dosed at 10 uM and 50 uM, respectively. Phosphonoformic acid (PFA; P6801; Sigma, St. Louis, MO, USA), a weak competitive inhibitor of *Pit1* transport activity, was used at a final concentration of 5 mM.

### RNAi ablation studies

C2C12 cells were seeded at 20,000/well and transfected with 20 nM of ON-TARGETplus siRNA (*Slc20a1 (Pit1*, L-045288–01–0005); GE Dharmacon, Lafayette, CO, USA) using RNAiMAX (13778075; Lipofectamine, Thermo Fisher Scientific, Waltham, MA, USA) diluted in Opti-MEM (31985070; Gibco, Gaithersburg, MD, USA) for delivery. Transfected cells were cultured in Pi-free DMEM for 3 days before further analysis.

### Extracellular flux analysis

Cellular respiratory activity was measured using a Seahorse XFe24 Analyzer (Agilent Technologies, Santa Clara, CA, USA). For transfection and non-transfection assays, C2C12 myoblasts were seeded at 40,000 cells/well in Pi-free DMEM (pH 7.4; with 10% FBS and 10 mM HEPES) and cultured for 3 days. Cells were re-fed the day before the assay.

For intact cell assays, microplates were placed in a CO_2_ free incubator 45 min before the start of the assay to permit evaporation of CO_2_. Immediately prior to the assay, Pi-free medium was replaced with assay medium (pH 7.4; 8.3 g/l DMEM base (CM038305; Invitrogen, Carlsbad, CA, USA) with 12.5 mM glucose, 31 mM NaCl, 100 mM pyruvate, 2 mM Glutamax-I (35050–061; Gibco, Gaithersburg, MD, USA), and no Pi). Oxygen consumption rate (OCR) was measured in 8.5 min intervals. Mitochondrial respiratory activity was manipulated by serial injection of 10 mM sodium Pi, 1.25 uM oligomycin (an ATP synthase inhibitor), 100 uM 2, 4-dinitrophenol (a mitochondrial uncoupler), and 1 uM antimycin/1 uM rotenone (complex I and III inhibitors). The effect of Pi on intact cell OCR is termed as “acute response”, and the following injection of oligomycin and DNP as “maximal respiration”.

Coupling and electron flow assay protocols were adapted from Divakaruni *et al*.^76^, Salabei *et al*.^77^, and the manufacturer’s protocols. C2C12 cells were permeabilized with Seahorse Plasma Membrane Permeabilizer (PMP, 102504–100; Agilent Technologies, Santa Clara, CA, USA). Briefly, immediately before each assay, cells were washed once with mannitol/sucrose (MAS) buffer (pH 7.2; 220 mM mannitol, 70 mM sucrose, 10 mM KH_2_PO_4_, 5 mM MgCl_2_, 2 mM HEPES, 1 mM EGTA, and 4 mg/mL fatty acid free-bovine serum albumin) then immersed in MAS buffer containing 1 nM PMP. OCR was measured in 3 min intervals. Mitochondrial respiratory state 3 (ADP + /− Pi + substrate) for coupling assays was induced by injection of the following: 4 mM ADP, 10 mM sodium Pi, 10 mM pyruvate and 1 mM malate (CI), 10 mM succinate (CII), 10 mM glycerol-3-phosphate (G3P, CIII), 100 uM TMPD and 10 mM ascorbic acid (CIV). State 4^o^ (oligomycin-dependent), maximal, and non-mitochondrial respiration were assessed by injection of chemicals mentioned above for intact cells. Electron flow assays were begun with cells immersed in MAS buffer containing PMP, DNP, and complex I-specific substrates, and additional complex-specific substrates were injected in serial order at the concentrations stated above.

### Statistical and image analysis

Data are expressed as means ± SEM and were analyzed as previously described^62^ in Prism 8.0 (GraphPad Software, Inc., La Jolla, CA). Differences between groups were considered significant if p-values obtained with Student’s t-test were < 0.05. The Mann-Whitney U test was used for comparisons when there was evidence by the Shapiro-Wilk normality test that the data were not normally distributed. Two-way ANOVA and Tukey’s test for multiple comparisons was used to determine significant differences between more than two treatment groups with a significance threshold of p < 0.05. Light and electron microscopic images were analysed using ImageJ (version2.0.0-rc-69/1.52p) to determine myofiber, myofibril (sarcomere) and mitochondrial areas.

## Supplementary information

Supplementary Information.

Supplementary Information 2.
