# Supplementary material for: Slc20a1/Pit1 and Slc20a2/Pit2 are essential for normal skeletal myofiber function and survival
Source: Sci Rep. 2020 Feb 20;10:3069. doi: 10.1038/s41598-020-59430-4 (PMC7033257; doi:10.1038/s41598-020-59430-4)

Supplemental Figures and Tables for: ***Slc20a1/Pit1* and *Slc20a2/Pit2* are essential for normal skeletal myofiber function and survival**

Sampada Chande, Daniel Caballero, Bryan B. Ho, Jonathan Fetene, Juan Serna, Dominik Pesta, Ali Nasiri, Michael Jurczak, Nicholas W. Chavkin, Nati Hernando, Cecilia M. Giachelli, Carsten A. Wagner, Caroline Zeiss, Gerald I. Shulman, Clemens Bergwitz

Table S1: Primers

ID	Gene	*	sequence
819	*Pit-1 ex2*	F	CTCATCCTGGGCTTCATCAT
820	*Pit-1 ex3*	R	CGGAAGCTTCAAAAACGAAG
822	*Pit-1 int4*	R	TTCCTTCCTGAATGCCCTCT
823	*CreY-1 F*	F	TGCCACGACCAAGTGACAGCAATG
824	*CreY-1 R*	R	AGAGACGGAAATCCATCGCTCG
954	*Pit-2 5'*	F	TAGATATGGAGGAATGAGAGCAGC
955	*Pit-2 3'*	R	CCATGCGTCAGAGCTCAGGAATCC
179	*Beta actin*	F	GGCTGTATTCCCCTCCATCG
180	*Beta actin*	R	CCAGTTGGTAACAATGCCATGT
843	*Beta actin (RT)*	F	GTGCTATGTTGCTCTAGACTTCG
844	*Beta actin (RT)*	R	GATGCCACAGGATTCCATACCC
838	*Pit1*	F	TGTATTGTCGGTGCAACCAT
839	*Pit1*	R	ATACCAGAAAGCAGCGGAGA
841	*Pit2*	F	ACCATTCGGAAAGGCATCATT
842	*Pit2*	R	GAAGGACGCGATCAACTGC
995	*Pit2Δex4*	F	CCACTTCTTTCTGGCTTCATGTCC
996	*Pit2Δex4*	R	TGAACTGATGGCGAGCTCAGACC
774	*Xpr1*	F	TCTGAGACTGAGGCTGTGGT
775	*Xpr1*	R	AGTAGTCCATGCTGGTGCAG
1001	*Myogenin*	F	CGATCTCCGCTACAGAGGC
1002	*Myogenin*	R	GTTGGGACCGAACTCCAGT
847	*Mfn2*	F	CCAACTCCAAGTGTCCGCTC
848	*Mfn2*	R	GTCCAGCTCCGTGGTAACATC
849	*Fis1*	F	AGAGCACGCAATTTGAATATGCC
850	*Fis1*	R	ATAGTCCCGCTGTTCCTCTTT
851	*Myh4*	F	CCGCATCTGTAGGAAGGGG
852	*Myh4*	R	GTGACCGAATTTGTACTGAGTGT
853	*Myh7*	F	GCTACGCTTCCTGGATGATCT
854	*Myh7*	R	CCTCTTAGTGTTGACAGTCTTCC
855	*Slc25a3*	F	TGATTGGCTATTCCATGCAAGG
856	*Slc25a3*	R	TCAAGGTGTTGGCATAACCAG
877	*Pgc1α*	F	AAGTGGTGTAGCGACCAATCG
878	*Pgc1α*	R	AATGAGGGCAATCCGTCTTCA
999	*Pax7*	F	TCTCCAAGATTCTGTGCCGAT
1000	*Pax7*	R	CGGGGTTCTCTCTCTTATACTCC
1030	*Pgk1*	F	ATGTCGCTTTCCAACAAGCTG
1031	*Pgk1*	R	GCTCCATTGTCCAAGCAGAAT
863	*Pkm1/2*	F	GCCGCCTGGACATTGACTC
864	*Pkm1/2*	R	CCATGAGAGAAATTCAGCCGAG
924	*Tfam*	F	AACACCCAGATGCAAAACTTTCA
925	*Tfam*	R	GACTTGGAGTTAGCTGCTCTTT
1038	*Xbp1*	F	GAACCAGGAGTTAAGAACACG

**1039**	***Xbp1***	**R**	AGGCAACAGTGTCAGAGTCC
**1016**	***CHOP***	**F**	GCGACAGAGCCAGAATAACA
**1017**	***CHOP***	**R**	GATGCACTTCCTTCTGGAACA
**1018**	***Fbxo32***	**F**	ACAAAGGAAGTACGAAGGAGCG
**1019**	***Fbxo32***	**R**	GGCAGTCGAGAAGTCCAGTC
**1022**	***Mt-Cytb***	**F**	TGAGGGGGCTTCTCAGTAGA
**1023**	***Mt-Cytb***	**R**	TAGGGCCGCGATAATAAATG
**310**	***Npt1***	**F**	CTGTCTTCTTTGGTATGGTCGTG
**311**	***Npt1***	**R**	ACAATGACTAAAGCAGCTCCG
**308**	***Npt3***	**F**	TCAACACCCTAGACGTTGCC
**309**	***Npt3***	**R**	GGAATCCGGTGGTAGTGGATG
**306**	***Npt4***	**F**	TGGAATCGCCTTCGTCACAC
**307**	***Npt4***	**R**	GTAGAGCTATTGAGGTGGGGT
**425**	***Slc17a4***	**F**	ACTTGAGCTTTGCCATAACCG
**426**	***Slc17a4***	**R**	TGCAGTGTTTCATTCCAGACAT
**429**	***Slc17a5***	**F**	CTGCATTGCCTTATTTTGGCTG
**430**	***Slc17a5***	**R**	CCAACCATTCCTACGAGGCTAA
**431**	***Slc17a6***	**F**	TGGAAAATCCCTCGGACAGAT
**432**	***Slc17a6***	**R**	CATAGCGGAGCCTTCTTCTCA
**302**	***Slc17a7***	**F**	ATGGCAGCTTCGGGATCTTTT
**303**	***Slc17a7***	**R**	CCTCAATGTATTTGCGCTCCTC
**498**	***Slc17a8***	**F**	TTCCCGGTGGCTTCATTTCAA
**499**	***Slc17a8***	**R**	CGCCGCAGAAGGGATAAACA
**1028**	***TATA***	**F**	AGAACAATCCAGACTAGCAGCA
**1029**	***TATA***	**R**	GGGAACTTCACATCACAGCTC
**1036**	***18S rRNA***	**F**	GCAATTATTCCCCATGAACG
**1037**	***18S rRNA***	**R**	GGCCTCACTAAACCATCCAA

*F=forward, R=reverse

<u>Table S2:</u> Anatomic phenotyping

Histo #	Genotype	Toe/ ear mark	Sex	Age (PN day)	Body weight (g)	Blood glucose	Radiographs	Milk spot	S-Creatinine
15	*smPit1*$^{-/-}$*;smPit2*$^{-/-}$	Z32	F	1	1.22g	ND	Yes	Yes	ND
16	*smPit1*$^{-/-}$*;smPit2*$^{-/-}$	Z33	M	1	0.97g	ND	Yes	Yes	ND
17	WT	Z34	F	1	1.24g	ND	Yes	Yes	ND
18	WT	Z35	M	1	1.31g	ND	Yes	Yes	ND
	WT	Z36	M	1	1.30g	ND	Yes	Yes	ND
	WT	Z37	F	1	1.30g	ND	Yes	Yes	ND
		Z38	M	1	1.38g	ND	Yes	Yes	ND
	WT	Z26	M	4	3.14g	ND	Yes	Yes	ND
	WT	Z27	F	4	3.4g	ND	Yes	Yes	ND
		Z28	F	4	2.96g	ND	Yes	Yes	ND
21	WT	Z29	F	4	2.9g	ND	Yes	Yes	ND
19	*smPit1*$^{-/-}$*;smPit2*$^{-/-}$	Z30	F	4	2.19g	ND	Yes	Yes	ND
20	*smPit1*$^{-/-}$*;smPit2*$^{-/-}$	Z31	F	4	2.03g	ND	Yes	Yes	ND
	WT	Z19	M	4	3.5 g	ND	ND	Yes	ND
	WT	Z20	F	4	3.4 g	ND	ND	Yes	ND
	WT	Z21	F	4	2.8 g	ND	ND	Yes	ND
13	WT	Z22	F	4	2.9 g	ND	ND	Yes	ND
	WT	Z23	M	4	2.9 g	ND	ND	Small	ND
		Z24	M	4	2.4 g	ND	ND	No	ND
14	*smPit1*$^{-/-}$*;smPit2*$^{-/-}$	Z25	F	4	1.7 g	ND	ND	Yes	ND
1	WT	Z1	F	8	3.03 g	ND	ND	Yes	ND
2	WT	Z2	F	8	3.72 g	150 mg/dl	ND	Yes	ND
3	WT	Z3	F	8	4.34 g	136 mg/dl	Yes	Yes	ND
4	WT	Z7	F	8	3.35 g	128 mg/dl	ND	Yes	ND
5	*smPit1*$^{-/-}$*;smPit2*$^{-/-}$	Z6	M	8	2.99 g	140 mg/dl	ND	Yes	ND
6	*smPit1*$^{-/-}$*;smPit2*$^{-/-}$	Z8	M	8	2.67 g	121 mg/dl	ND	Yes	ND
7	*smPit1*$^{-/-}$*;smPit2*$^{-/-}$	Z9	M	8	2.40 g	139 mg/dl	ND	Yes	ND
8	*smPit1*$^{-/-}$*;smPit2*$^{-/-}$	Z10	F	8	2.46 g	122 mg/dl	Yes	Yes	ND
9	WT	Z11	F	10	6 g	ND	Yes	Yes	0.5 mg/dl
10	WT	Z14	M	10	6.88 g	ND	Yes	Yes	0.5 mg/dl

	smPit1$^{-/-}$;*smPit2*$^{-/-}$	Z15	M	10	2.26 g	ND	Yes	Small	0.5 mg/dl
	smPit1$^{-/-}$;*smPit2*$^{-/-}$	Z16	F	10	2.31 g	ND	Yes	Yes, large	0.5 mg/dl
11	*smPit1*$^{-/-}$;*smPit2*$^{-/-}$	Z17	F	10	1.95 g	ND	Yes	No	0.5 mg/dl
12	*smPit1*$^{-/-}$;*smPit2*$^{-/-}$	Z18	M	10	1.61 g	ND	Yes	Yes, large	0.5 mg/dl

Table S3: Mendelian inheritance

**Genotype**	**Expected males**	**Obtained males***	**Expected females**	**Obtained females***	**Indeterminate sex***
***Pit1*$^{fl/fl}$;*Pit2*$^{fl/+}$**	12.5	10.15 (14)	12.5	10.15 (14)	
***smPit1*$^{-/-}$;*smPit2*$^{-/+}$**	12.5	10.15 (14)	12.5	8.69 (12)	
***Pit1*$^{fl/fl}$;*Pit2*$^{fl/fl}$**	12.5	14.5 (20)	12.5	13.04 (18)	
***smPit1*$^{-/-}$;*smPit2*$^{-/-}$**	12.5	13.8 (19)	12.5	5.07 (7)	
**Dead/Ungenotyped / Missing pups**		2.17 (3)		1.45 (2)	10.87 (15)

Mating strategy: Male (*smPit1*$^{-/-}$;*smPit2*$^{-/+}$) with female (*Pit1*$^{fl/fl}$;*Pit2*$^{fl/fl}$); *Percentage (Number of mice)

**Genotype**	**Expected males**	**Obtained males***	**Expected females**	**Obtained females***	**Indeterminate sex***
***Pit1*$^{fl/+}$;*Pit2*$^{fl/fl}$**	12.5	9.30 (8)	12.5	9.30 (8)	
***smPit1*$^{-/+-}$;*smPit2*$^{-/-}$**	12.5	18.60 (16)	12.5	17.44 (15)	
***Pit1*$^{fl/fl}$;*Pit2*$^{fl/fl}$**	12.5	4.65 (4)	12.5	11.62 (10)	
***smPit1*$^{-/-}$;*smPit2*$^{-/-}$**	12.5	10.46 (9)	12.5	9.30 (8)	
**Dead/Ungenotyped / Missing pups**				1.16 (1)	8.13 (7)

Mating strategy: Male (*smPit1*$^{-/+}$;*smPit2*$^{-/-}$) with female (*Pit1*$^{fl/fl}$;*Pit2*$^{fl/fl}$); *Percentage (Number of mice)

Video V1: Four *smPit1*$^{-/-}$*;smPit2*$^{-/-}$ mice showed weight loss and progressively diminishing mobility, with severe ambulatory decline by P10 when compared to one (larger) WT littermate.

Figure S1: Semi-quantitative RT-PCR analysis of Pi transporters in WT mouse skeletal muscle

A: Semi-quantitative RT-PCR showing relative expression levels of Pi transporters in C2C12 cells, mouse and human skeletal muscle (data for human skeletal muscle were obtained from Nishimura et al. Drug Metab. Pharmacokinet. 23 (1): 22–44 (2008)). **B:** Semi-quantitative RT-PCR estimating relative expression for *Pit1* and *Pit2* in different WT mouse tissues. **C:** Genotyping PCR for the recombinant allele of $Pit1^{delta3,4}$ *and* $Pit2^{delta4}$ in quadriceps and hearts of seven different $smPit1^{-/-};smPit2^{-/-}$ mice at P10. For raw images see below. **D:** Semi-quantitative RT-PCR showing equal expression of *mPit1* and *mPit2* in WT and $smPit1^{-/-};smPit2^{-/-}$ mice.

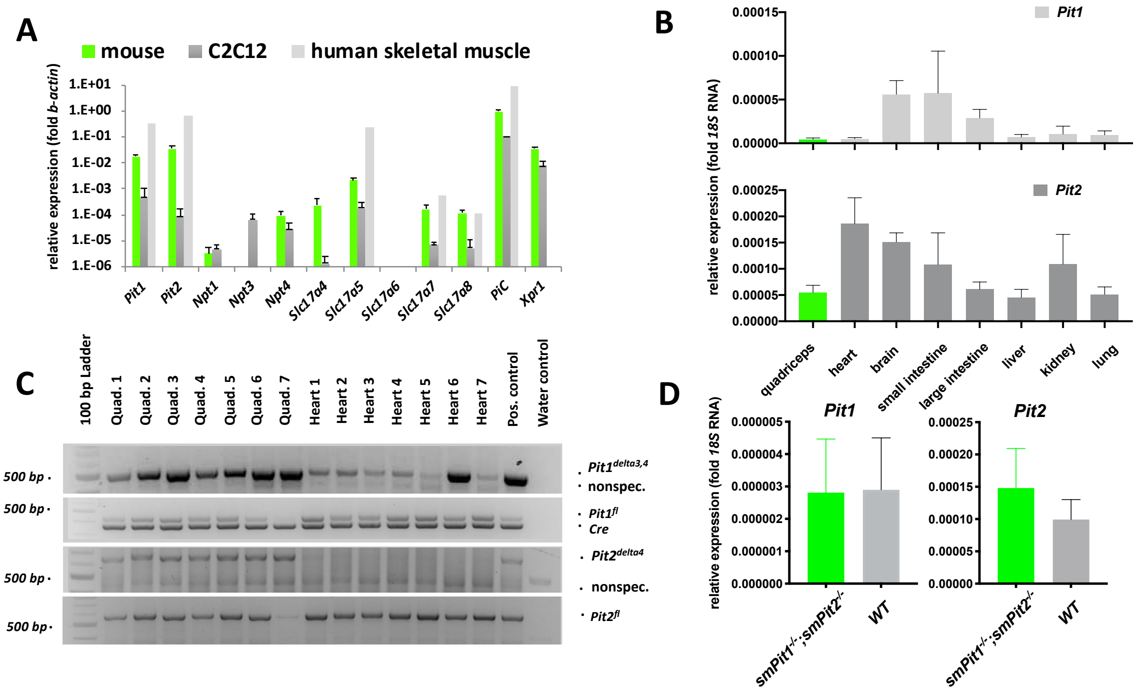

Figure S2: Complete histological analysis of *smPit1$^{-/-}$;smPit2$^{-/-}$* mice

A: Muscle phenotype, epaxial muscle, at P1, P4 and P10. Compared to the WT animal (***a, c, e***), myofibers in the mutant (***b, d, f***) are thinner with greater interstitial cellularity and larger intercellular spaces (arrows, ***f***). H&E, bar = 50 µm. **B:** Muscle phenotype, diaphragm, at P1, P4 and P10. Compared to WT (***a, c, e***), myofibers in *smPit1$^{-/-}$;smPit2$^{-/-}$* mice (***b, d, f***) exhibited comparable width and organization. H&E, bar = 50 µm. **C:** Proximal tibia and lumbar vertebrae, P10, sagittal section. ***a, b***: Proximal tibial epiphysis. Compared to WT (***a***), long bones in *smPit1$^{-/-}$;smPit2$^{-/-}$* mice (***b***) were smaller and less mature. ***c, d***: Proximal tibial epiphysis. Both WT (***c***) and *smPit1$^{-/-}$;smPit2$^{-/-}$* mice (***d***) retained comparable epiphyseal architecture, however mutant chondrocytes had less cytoplasmic volume. The correspondingly smaller hypertrophic zone (asterisk) and shorter, less robust primary bone spicules (arrow) are illustrated (***b***). ***e, f***: Lumbar vertebrae. Although the bone was proportionately smaller in *smPit1$^{-/-}$;smPit2$^{-/-}$* mice (***f***), its structural components were comparable to WT (***e***). H&E, Bar =200 µm (***a, b, e, f***), 50 µm (***c, d***). **D:** Interscapular region, P10, sagittal section. In contrast to WT (***a***), *smPit1$^{-/-}$;smPit2$^{-/-}$* mice (***b***) displayed almost complete loss of subcutaneous white adipose tissue (WAT) in the interscapular region, as well as in the dermis (arrow). Brown adipose tissue (BAT) was retained. H&E, Bar =500 µm (***a***), 200 µm (***b***). **E:** General organ histology in WT and *smPit1$^{-/-}$;smPit2$^{-/-}$* mice, P10. ***a-d***: Stomach and intestine. Although the stomach of individual *smPit1$^{-/-}$;smPit2$^{-/-}$* and WT mice contained milk (***a, b***), *smPit1$^{-/-}$;smPit2$^{-/-}$* mice displayed smaller intestinal loops (***b***) and smaller villi (***d***), indicative of sustained lack of trophic support. ***e, f***: Heart and lung. The generalized smaller size of *smPit1$^{-/-}$;smPit2$^{-/-}$* mice (***f***) was reflected in smaller thoracic viscera compared to WT (***e***), with retention of normal cardio-pulmonary histology. (Apparent difference in cardiac anatomy is created by differing planes of section). ***g, h***: Skin, interscapular region. Dermal (red arrows) and hypodermal (black arrows) white adipose tissue was entirely lost in *smPit1$^{-/-}$;smPit2$^{-/-}$ mice* (***h***) compared to WT (***g***) animal. Follicular anatomy was comparable. *smPit1$^{-/-}$;smPit2$^{-/-}$* mice displayed mild orthokeratotic hyperkeratosis corresponding to flaky greyish appearance grossly. ***i, j***: Liver. Hepatic architecture was comparable in WT (***i***) and *smPit1$^{-/-}$;smPit2$^{-/-}$* mice (***j***) mice. ***k, l***: Kidney. Renal size was smaller, and tubule-glomerular development less advanced in the mutant (***l***), compared to the WT (***k***) animal. H&E, bar = 500 µm (***a, b, e, f***); 200 µm (***g, h***); 100 µm (***I, j***); 50 µm (***k, l***).

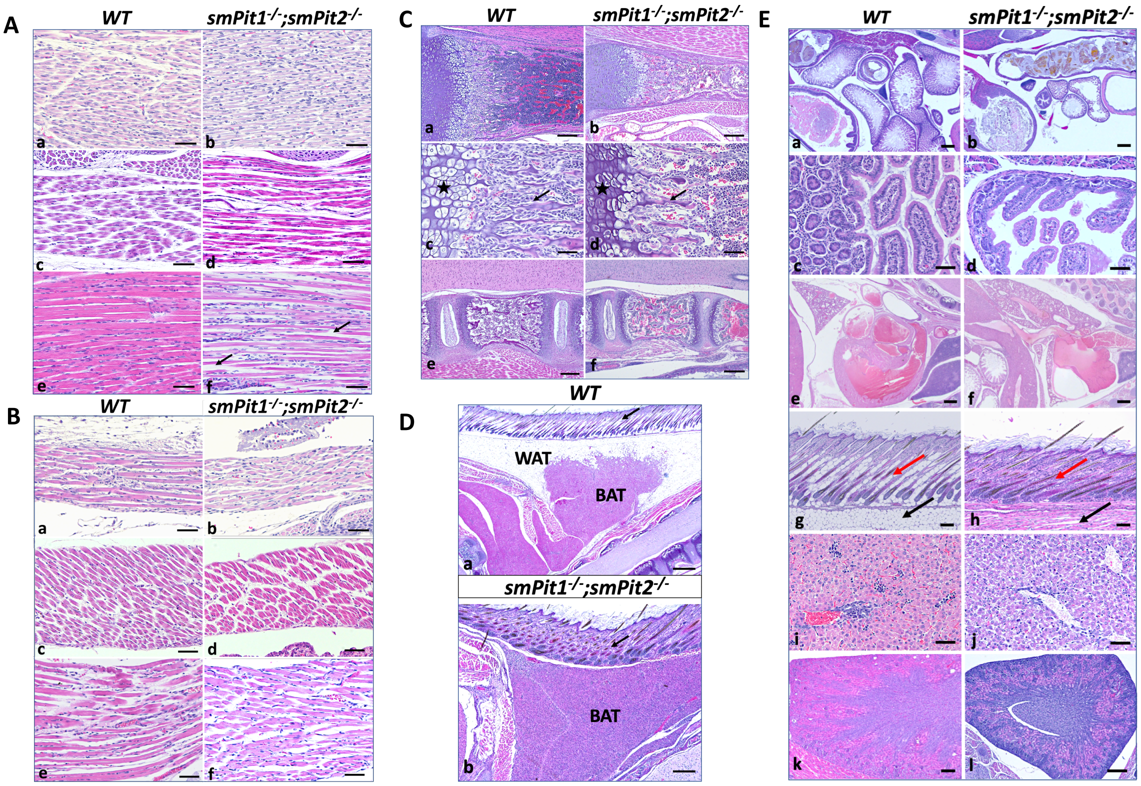

A
WT
smPit1-/-;smPit2-/-
a
b
c
d
e
f
B
WT
smPit1-/-;smPit2-/-
a
b
c
d
e
f
C
WT
smPit1-/-;smPit2-/-
a
b
c
d
e
f
D
WT
WAT
BAT
a
smPit1-/-;smPit2-/-
BAT
b
E
WT
smPit1-/-;smPit2-/-
a
b
c
d
e
f
g
h
i
j
k
l

Figure S3: No evidence for rhabdomyolysis, ER-stress or proteasomal activation in DKO quadriceps and gastrocnemius muscles

A: Evans blue was injected i.p. at P10. WT and *smPit1*$^{-/-}$;*smPit2*$^{-/-}$ mice were sacrificed after 24 hrs., and intracellular signal intensity in frozen sections of quadriceps muscle was evaluated by fluorescent confocal microscopy. No difference in semi-quantitative RT-PCR of ER-stress markers X-box binding protein 1 (*Xbp1*) splicing (**B**) and CCAAT/Enhancer-Binding Protein Homologous Protein (*Chop*, **C**), the proteasomal marker muscle-specific ubiquitin ligase F-box protein 32 (*Fbxo32*, **D**), or of absolute expression for the house-keeping genes *beta actin* (**E**) and *18S RNA* **(F)** when normalized over sample RNA was observed. **G:** TEM of gastrocnemius muscle showed normal rough endoplasmic reticulum (arrows) and absence of autophagosomes in *smPit1*$^{-/-}$;*smPit2*$^{-/-}$ mice at P10.

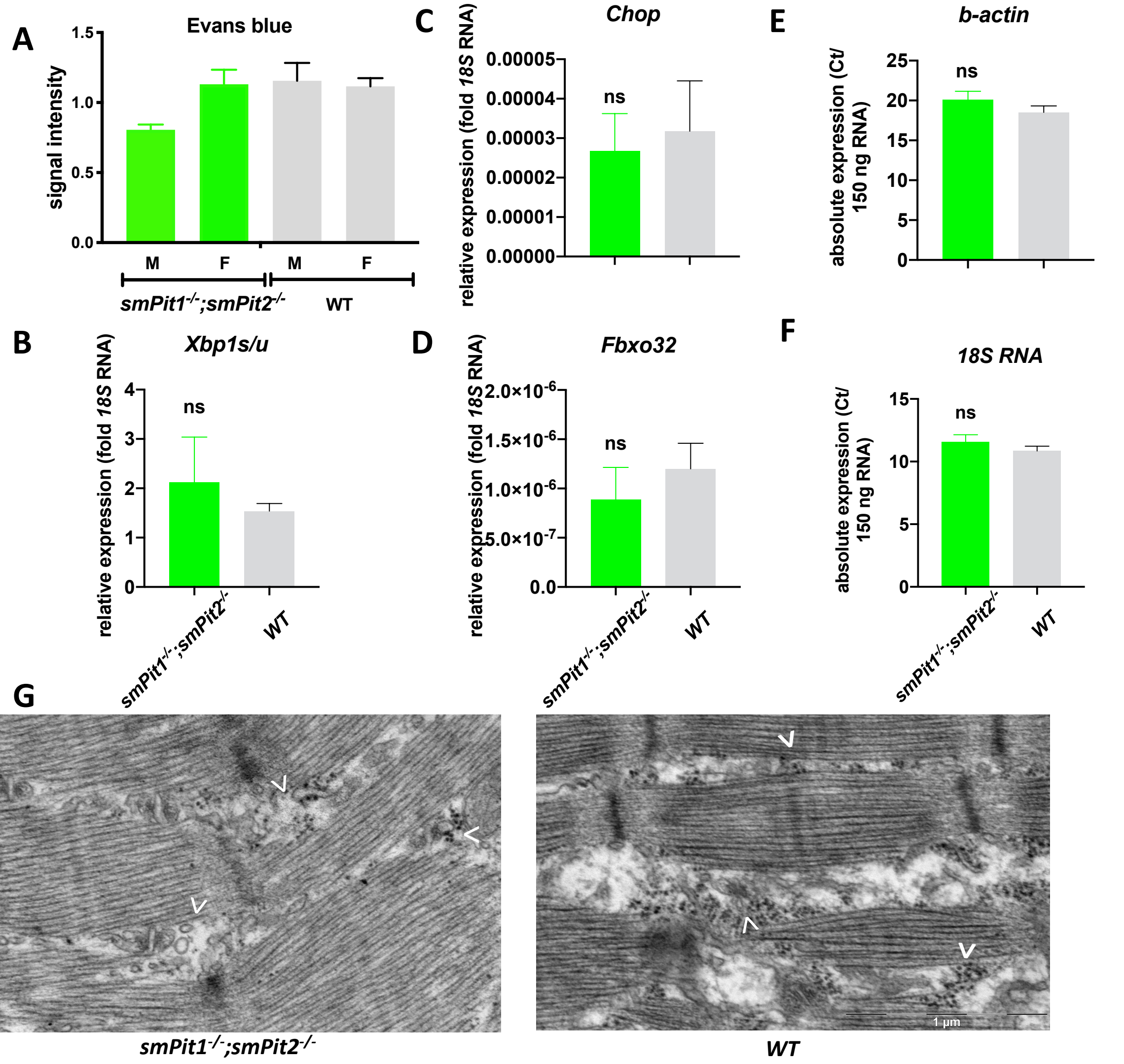

Figure S4: Mitochondrial pathology

A: immunoblot used for quantification in Fig. 4D showing increased pAMPK/tAMPK ratio and reduced pERK1/2/tERK1/2 ratio in quadriceps of four different *smPit1*$^{-/-}$;*smPit2*$^{-/-}$ mice at P10. For raw images see below. **B:** Semi-quantitative genomic PCR analysis of mitochondrial cytochrome b (mt-Cytb) corrected for nuclear beta actin showed normal mitochondrial mass in *smPit1*$^{-/-;}$*smPit2*$^{-/-}$ mice at P10. Means±SEM, n=5-8, $^{****}p<0.00002$, $^{***}p=0.0002$, $^{**}p=0.002$, $^{*}p=0.03$ vs. WT. **C-F:** TEM showing distended mitochondria (white arrows) with matrix lucencies in cross sections and longitudinal sections of *smPit1*$^{-/-}$;*smPit2*$^{-/-}$ mice at P10.

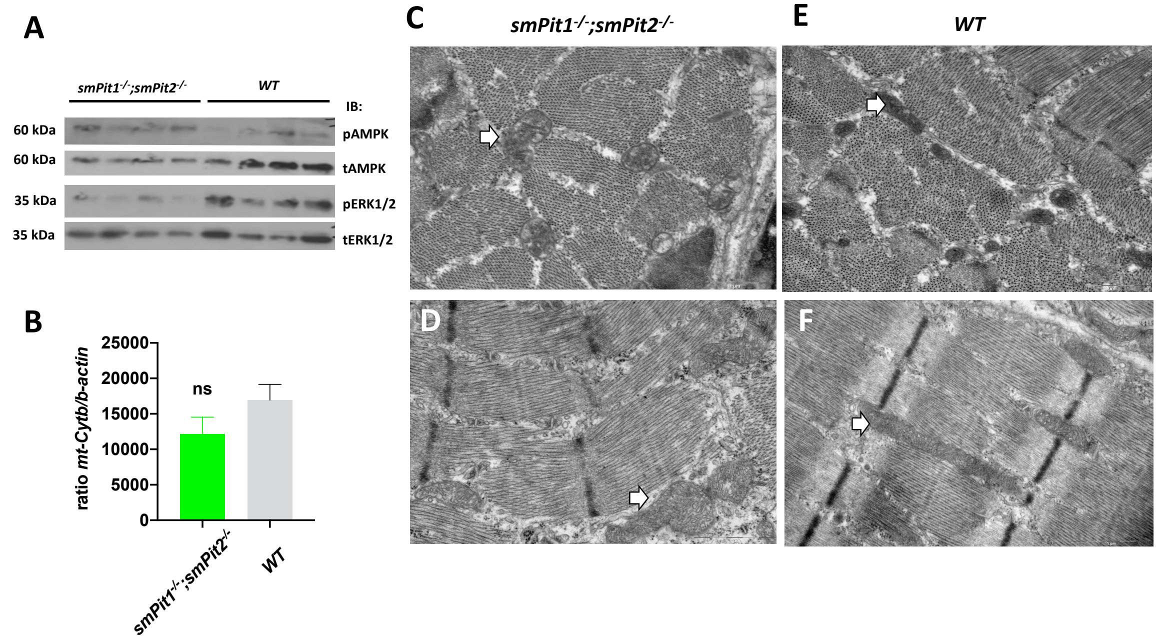

Figure S5: siRNA mediated ablation of *mPit1* and *mPit2* blocks activation of pERK1/2 in C2C12 cells

A: Summary of several immunoblots used to measure Pi-induced activation of pERK1/2 in lysates obtained from C2C12 cells treated with 10 mM Pi (P10) for 5 min., following knockdown of *mPit1* and *mPit2*. **B:** Knockdown was verified using semi-quantitative RT-PCR of total RNA prepared from the same cells. Means±SEM, n=5-8, ****p<0.00002, ***p=0.0002, **p=0.002, *p=0.03 vs. control/P0, $^{\#}$p=0.03 vs. P10

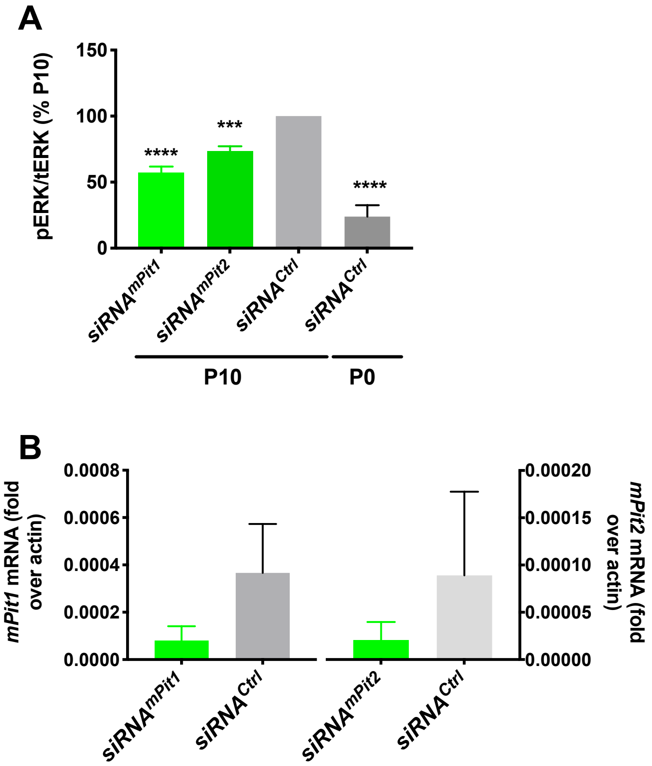

Figure S6: Grip strength is unaffected in single transporter and three-allele mutants at P60.

A: Grip strength improved with age and was lower for female than males. **B:** Although significant between 4 week *WT* and *HSA-Cre 1* females ($), and 16 week *WT1* and *HSA-Cre 1* males (#), the difference observed for weights was smaller between genotypes than the difference seen between gender and age. All mice thrived normally, were fertile, and had comparable body weights **C**: Grip strength and **D**: weights were similar between genotypes, except for a small weight difference between *WT1* and *WT2* males. Shown are means+/-SEM, n=5-12, ****p<0.00002, ***p=0.0002, **p=0.002, *p=0.03 vs. WT.

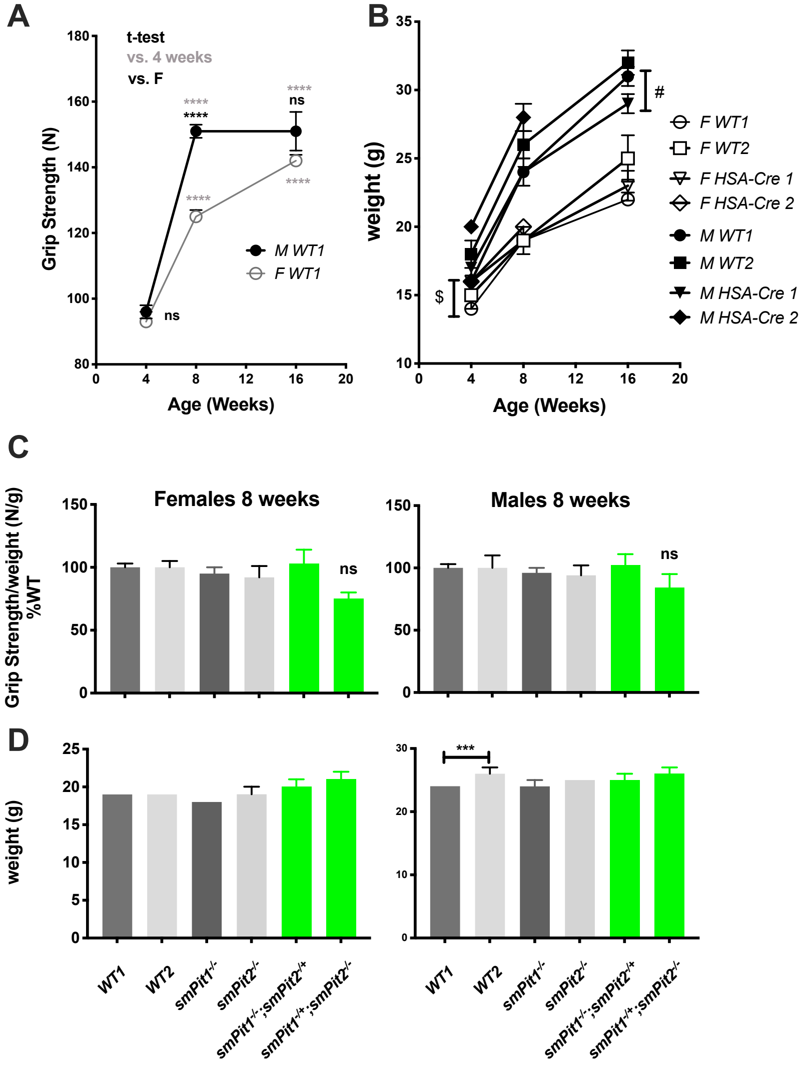

Raw Figure 1B: Genotyping PCR for *Pit1*^{fl}, *HSA-Cre*, and *Pit1*^{wt}.

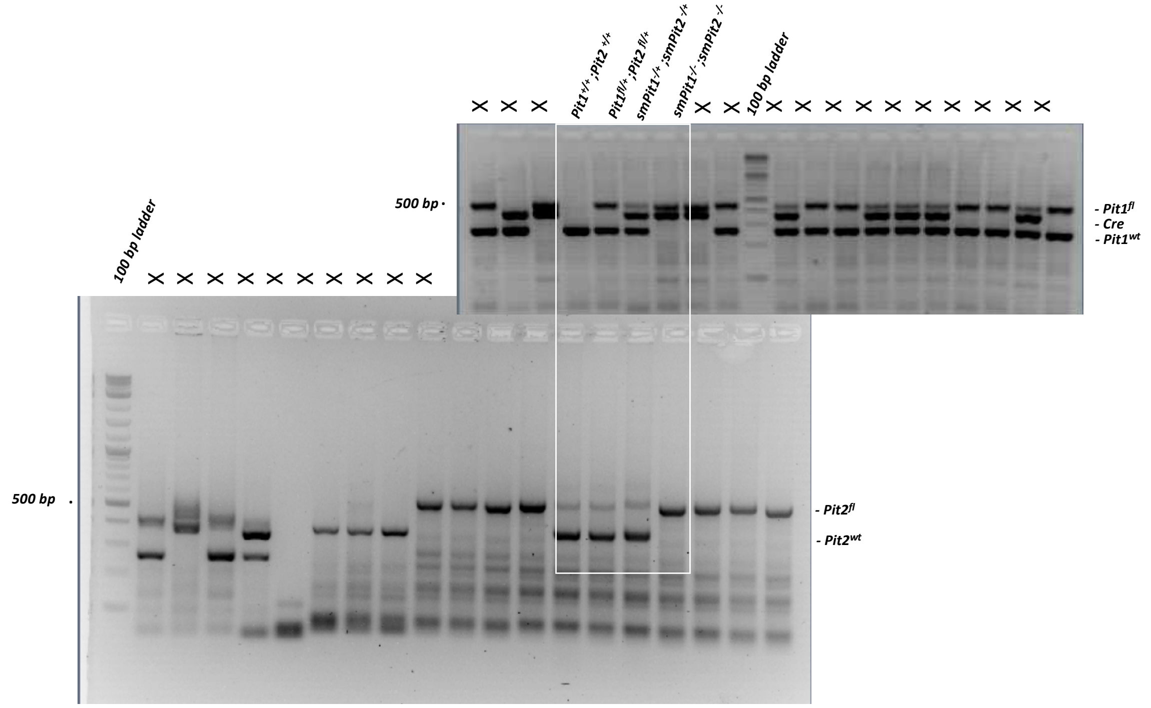

Raw Figure 1C, top two panels: Genotyping PCR for the recombinant *Pit1*^{delta3,4} and *Pit2*^{delta4} in various tissues of a *smPit1*$^{-/-}$*;smPit2*$^{-/-}$ mouse at P10.

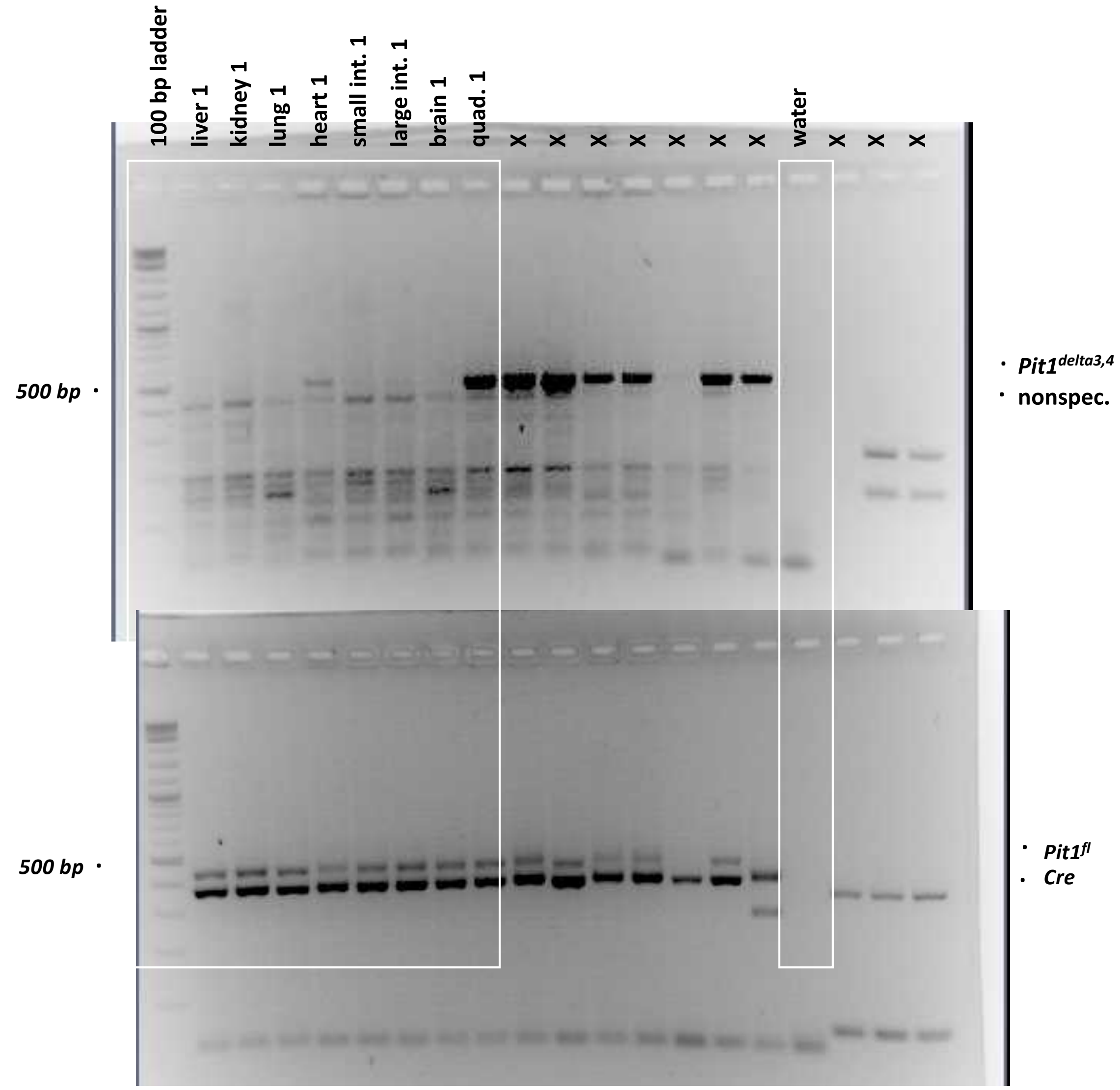

Raw Figure 1C, bottom two panels: Genotyping PCR for the recombinant *Pit1*^{delta3,4} and *Pit2*^{delta4} in various tissues of a *smPit1*$^{-/-}$;*smPit2*$^{-/-}$ mouse at P10.

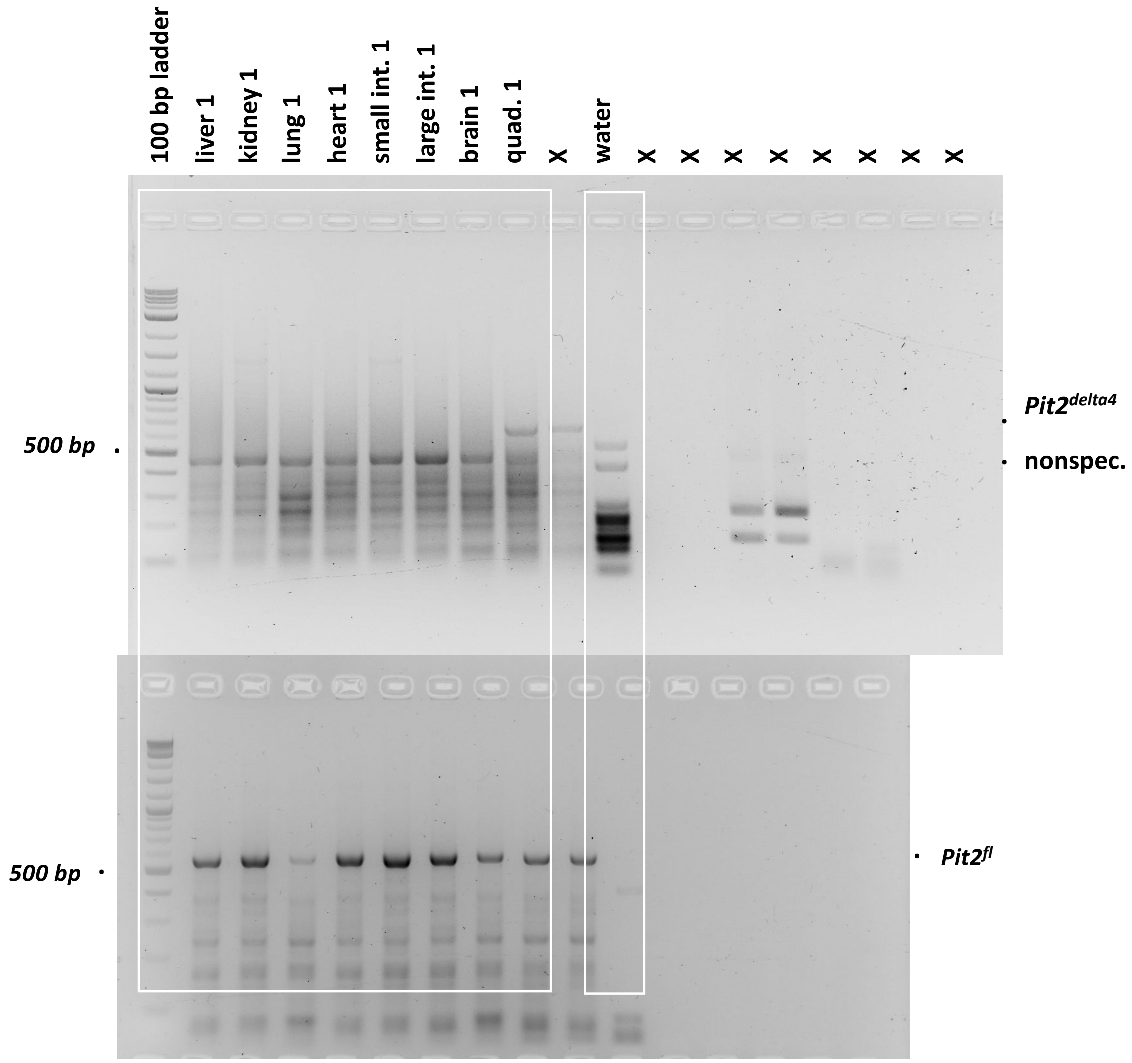

Raw Figure S1C: Genotyping PCR for the recombinant allele of *Pit1*^{delta3,4} *and Pit2*^{delta4} in quadriceps and hearts of seven different *smPit1*$^{-/-}$;*smPit2*$^{-/-}$ mice at P10.

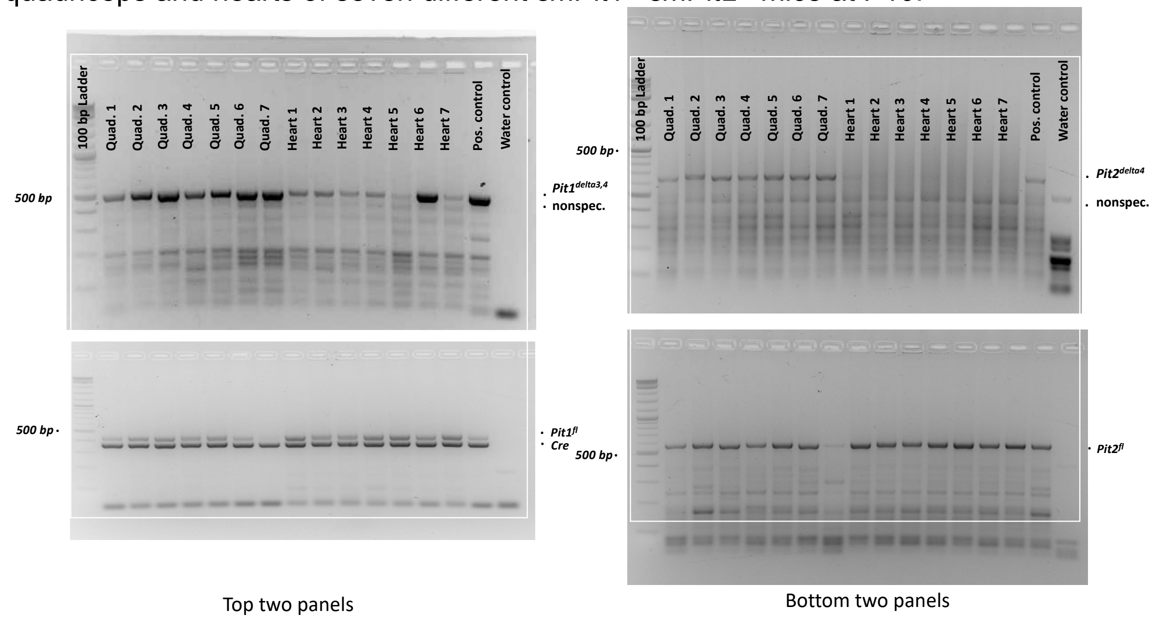

Raw Figure S4A: immunoblot used for quantification in Fig. 4D showing increased pAMPK/tAMPK ratio and reduced pERK1/2/tERK1/2 ratio in quadriceps of four different *smPit1*$^{-/-}$;*smPit2*$^{-/-}$ mice at P10.

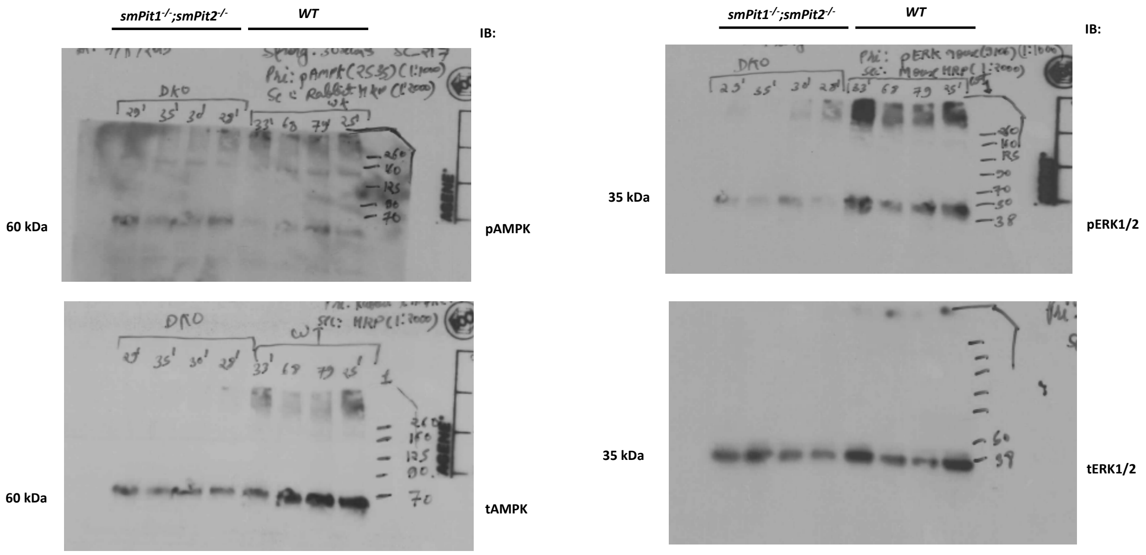

Supplement: Supplementary file 2 — Supplementary Information 2. [file 41598_2020_59430_MOESM2_ESM.pdf]
